# Raman Spectroscopy of Fullerenes: From C_60_ to Functionalized Derivatives

**DOI:** 10.3390/molecules30030738

**Published:** 2025-02-06

**Authors:** Yifan Qin, Jilian Xu, Zhewen Liang, Haijun Teng, Da Zhan, Hai Xu

**Affiliations:** 1 State Key Laboratory of Luminescence Science and Technology, Changchun Institute of Optics, Fine Mechanics and Physics, Chinese Academy of Sciences, Changchun 130033, China; qinyifan20@mails.ucas.ac.cn (Y.Q.); liangzw96@163.com (Z.L.); tenghaijun19@mails.ucas.ac.cn (H.T.); zhanda@ciomp.ac.cn (D.Z.); xuhai@ciomp.ac.cn (H.X.); 2 University of Chinese Academy of Sciences, Chinese Academy of Sciences, Beijing 100049, China

**Keywords:** fullerene, fullerene derivatives, Raman, SERS, TERS

## Abstract

Fullerenes, a unique allotrope of carbon, have captured significant attention in multiple scientific fields. As a non-destructive characterization technique, Raman spectroscopy has proven indispensable for investigating fullerenes and their derivatives, offering detailed insights into their vibrational properties. This review discusses the broad utility of Raman spectroscopy in revealing the structural and physicochemical characteristics of fullerenes—from the iconic C_60_ molecule to an array of its derivatives—highlighting its capacity to detect functionalization-induced changes in molecular structure and electronic properties, while also assessing environmental influences such as solvent effects and temperature variations. Particular emphasis is placed on advanced Raman-based techniques, including enhanced Raman spectroscopy, surface-enhanced Raman spectroscopy (SERS), and tip-enhanced Raman spectroscopy (TERS), for the characterization of fullerenes and their derivatives. These cutting-edge methods offer high sensitivity and ultra-high spatial resolution, greatly expanding the scope of fullerene research and delivering deeper insights into their structural and functional properties.

## 1. Introduction

Since its discovery in 1985 [1], fullerene, a carbon allotrope, has attracted considerable interest in the scientific community due to its stable chemical properties, distinctive physical characteristics (such as high electrical and thermal conductivity, along with excellent nonlinear optical properties), and environmental friendliness [2,3,4,5,6,7,8,9,10,11]. It has been extensively applied across a wide range of fields, including energy, agriculture, superconductors, catalysis, mechanical engineering, life sciences, and materials science [12,13,14,15,16,17,18,19,20,21]. The most notable fullerene is the C_60_ molecule, recognized for its hollow, cage-like structure made up of 12 pentagonal and 20 hexagonal carbon rings [22]. As research has progressed, fullerenes with varying carbon atom counts, from C_20_ to those with up to 300 carbon atoms, have been synthesized, greatly expanding the fullerene family [23,24,25,26]. These fullerene molecules exhibit diverse structural configurations and distinct physical and chemical properties, offering greater scope and flexibility for extensive applications.

**Figure 1:**
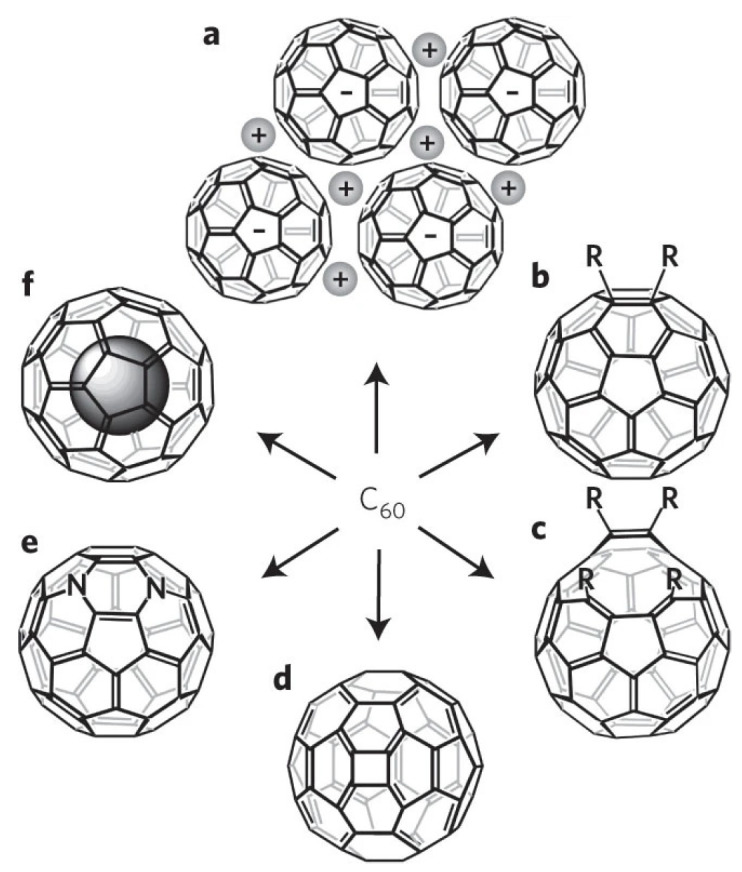
Fullerene derivatives classification: (a) fullerene salts, (b) functionalized fullerenes, (c) open−cage fullerenes, (d) quasi-fullerenes, (e) heterofullerenes, (f) endohedral fullerenes. Reproduced with permission from [49]. N represents nitrogen atom for example, and R represents functional groups. Copyright 2010 Springer Nature Limited.

Recently, research has extended to fullerene derivatives, such as endohedral fullerenes (Figure 1), which contain metal atom clusters or molecules embedded within the fullerene structure. These derivatives exhibit unique electronic and magnetic properties, promising broad applications in fields like biomedicine, photo-induced electron transfer, and quantum computing [27,28,29,30,31,32,33]. For example, water-soluble endohedral gadolinium fullerene derivatives Gd@C_60_(OH)_x_ and Gd@C_60_[C(COOH)_2_]_10_ show potential applications as MRI contrast agents. The metal Gd atoms are encapsulated in fullerene cages, making them safer to use in vivo [33]. Additionally, by modifying their surfaces with different functional groups, functionalized fullerenes can improve solubility, biocompatibility, and interactions with biological macromolecules, showing potential in biomedical applications such as drug delivery, agriculture, and photodynamic therapy [34,35,36,37,38,39,40,41,42]. C_60_(C(COOH)_2_)_2_ and C_60_(OH)_22_ have proved to be able to protect cells against H_2_O_2_-induced oxidative damage, stabilize the mitochondrial membrane potential, and reduce intracellular reactive oxygen species (ROS) production [38]. Through chemical linking or “cage-opening” methods, fullerene molecules can form one-dimensional chains or two-dimensional layers with specific structures and functions, possessing unique topological and optoelectronic properties crucial for molecular electronics, machines, and low-dimensional devices [43,44,45,46,47,48].

As research progresses, fullerenes and their derivatives are playing increasingly important roles in various fields of study. It is of vital importance to characterize the types and vibrational modes of chemical bonds in various fullerene derivatives, as it aids in exploring their structural, mechanical, and electrical properties. However, the vast diversity and complexity of fullerenes derivatives necessitate advanced characterization techniques to conduct characterization. Raman spectroscopy is a non-destructive optical characterization technique which sensitively reflects the vibrational characteristics of molecular groups and subtle environmental influences by analyzing changes in vibrational modes, making it an ideal tool for studying fullerenes and their derivatives. The unique spherical structure of fullerene molecules is reflected in their characteristic Raman peaks, providing crucial information about their molecular structure. Functionalization, such as the addition of organic groups or the encapsulation of atoms or molecules, induces notable shifts in the Raman spectrum, including the emergence of new peaks and alterations in existing ones, which directly correlate with changes in molecular structure and properties when organic functional groups are added, or molecules or atoms are embedded to create derivatives. Significant changes occur in the molecular structure and properties, leading to the emergence of new characteristic peaks and shifts in the original Raman spectrum [50]. Raman spectroscopy can also analyze the charge transfer process between the fullerene cage and organic functional groups or embedded molecules [51]. For fullerene derivatives widely used in the field of biomedicine, it is often necessary to perform non-destructive characterization in an aqueous solution environment. Raman spectroscopy is less affected by water solvents, and is the optimal method for characterizing vibration modes in this environment.

The inherently weak intensity of conventional Raman signals limits their effectiveness in providing precise characterization of molecular vibrational processes, particularly at low concentrations or on the nanoscale. Advanced Raman spectroscopy, such as resonance-enhanced Raman spectroscopy, surface-enhanced Raman spectroscopy (SERS), and tip-enhanced Raman spectroscopy (TERS), has significantly improved the detection sensitivity of fullerene derivatives, even achieving single-molecule spectral resolution [52,53]. This enhanced sensitivity is crucial for investigating molecular adsorption characteristics, vibrational modes, interfacial behaviors, and fine structural details. Furthermore, TERS allows for the probing of optical and vibrational heating effects at the nanoscale. These advanced Raman-based approaches thus greatly expand the scope of fullerene research, providing deeper insights into their structures and dynamics.

In this review, we have summarized key studies on conventional and advanced Raman spectroscopy for probing fullerenes and their derivatives, emphasizing its advantages in characterizing molecular structures, dynamic processes, and electron transfer properties.

## 2. Raman Principles and Fullerene Resonant Modes

**Figure 2:**
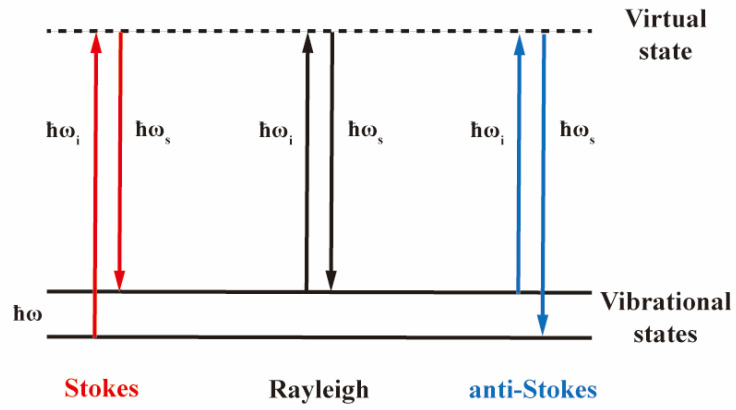
Schematic of energy transition processes in Rayleigh scattering and Raman scattering.

Raman spectroscopy, as an inelastic scattering technique, offers several notable advantages, including minimal sample consumption, high spatial resolution, detailed spectral information, non-destructive and contactless measurement, and user-friendly instrumentation. These attributes make it a powerful tool for studying lattice vibrations and chemical bond dynamics. When materials are exposed to laser light, both elastic scattering—such as Rayleigh scattering, where the incident photon energy (ħω_i_) equals the scattered photon energy (ħω_s_)—and inelastic scattering—such as Raman scattering, where ħω_i_ differs from ħω_s_—occur simultaneously (Figure 2). Raman scattering only occurs when the polarizability of the vibrating molecules or lattices changes during scattering process. In Raman scattering, the energy difference between the incident and scattered photons corresponds to the energy of vibrational or rotational energy levels (or the phonon energy of lattice vibrations), which is denoted as ħω. Stokes scattering corresponds to the situation when ħω_i_ − ħω = ħω_s_, representing energy transfer from photons to the material. Anti-Stokes scattering corresponds to ħω_i_ + ħω = ħω_s_, representing energy transfer from the material to photons. Raman peak positions act as a unique fingerprint spectrum for each material, as the material has characteristic vibrational energy levels, thus possessing a unique set of ħω values. Shifts in Raman peak positions indicate the changes in vibrational characteristics, which can further inform the alterations in molecular structure or electronic distribution. According to thermodynamic statistical laws, the distribution of electrons on energy levels follows the Boltzmann distribution, leading to a relationship between Stokes and anti-Stokes scattering intensities as I_S_/I_aS_ ~ exp(ħω/kT), making Raman scattering a valuable tool for measuring sample temperature.

**Figure 3:**
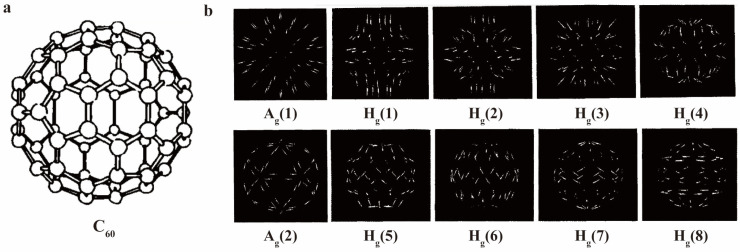
(**a**) Diagram of C_60_ molecular structure. (**b**) Schematic diagram of different C_60_ molecular vibration modes according to Raman modes. Reproduced with permission from [57]. Copyright 1996 John Wiley and Sons.

Fullerene materials, characterized by their three-dimensional structures composed of numerous carbon atoms, provide rich Raman spectral information. Among them, C_60_ and C_70_ are two typical representatives of fullerenes. C60 is a molecular cage of 0.71 nm in diameter, consisting of 60 carbon atoms, exhibiting Ih point group symmetry and featuring 10 Raman-active vibrational modes (2A_g_ + 8H_g_) (Figure 3) [54,55]. In contrast, C_70_ has an ellipsoidal molecular cage with D5h point group symmetry and possesses 53 Raman-active vibrational modes (12$A_{1}^{\prime }$ + 22$E_{2}^{\prime }$ + 19$E_{1}^{\prime \prime }$) [56]. Due to its lower molecular symmetry, C_70_ presents more intricate Raman information, making it challenging to capture all Raman signals accurately during experiments. Table 1 lists all experimentally determined Raman peaks for C_60_, including its characteristic A_g_ and H_g_ modes, and some captured peaks for C_70_, which are more numerous due to its lower symmetry. In the presence of endohedral molecules or attached functional groups, the Raman peak positions of fullerene molecules shift, accompanied by changes in peak width and intensity. These characteristics are fundamental to the Raman analysis of fullerene derivatives, which will be explored in detail in the following sections.

## 3. Raman Spectrometer Configuration

### 3.1. Conventional Raman Spectroscopy

**Figure 4:**
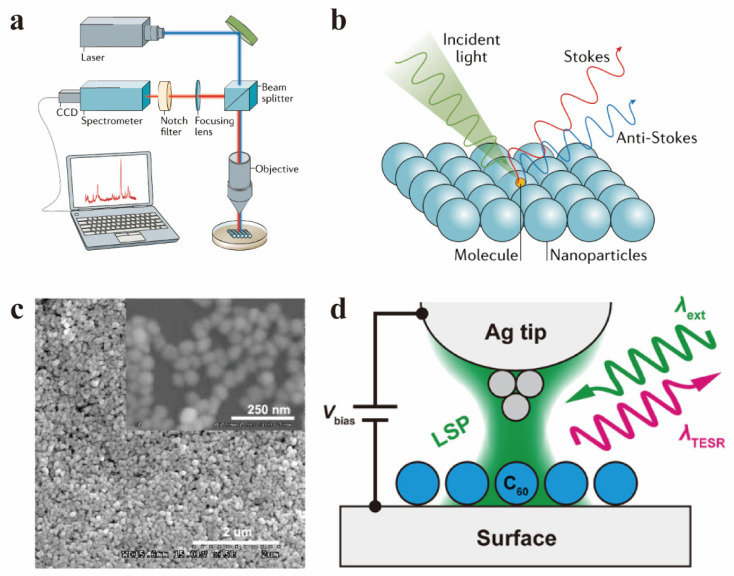
(**a**) A standard Raman spectrum setting with top illumination configuration. (**b**) Schematic diagram of the SERS with characterized molecule on metal nanoparticles. (**a**,**b**) Reproduced with permission from [63]. Copyright 2022 Springer Nature Limited. (**c**) An SEM image of the Au nanoparticle substrate prepared for SERS characterization. Reproduced with permission from [52]. Copyright 2011 John Wiley and Sons. (**d**) Schematic diagram of the TERS measurement. Reproduced with permission from [64]. Copyright 2022 American Chemical Society.

Conventional Raman spectroscopy typically consists of a laser light source, an optical system (including focusing, filtering, and dispersion systems), a detector, and a data processing system (Figure 4a) [62]. Common laser sources used for studying fullerene materials include argon ion lasers with working wavelengths of 488 nm and 514.5 nm, Nd:YAG lasers at 532 nm, He-Ne lasers at 633 nm, and semiconductor lasers at 785 nm. Among these, the 514.5 nm argon ion laser is most compatible with the electronic transition energy levels of fullerene materials, and is widely used in this field. Using a 488 nm or shorter wavelength laser provides higher photon energy, resulting in stronger Raman signals, but also produces stronger background fluorescence. On the other hand, lasers with longer wavelengths have lower photon energy, leading to weaker Raman signals but effectively suppressing background noise. For selective resonance enhancement of specific vibrational modes in fullerene materials, both short-wavelength and long-wavelength lasers have certain applications. Optimizing the optical path is crucial for improving the signal-to-noise ratio of Raman signals. Before Raman testing, the optical path must be collimated, to minimize signal loss. Additionally, selecting appropriate filters helps suppress background noise. The choice of grating is also critical: higher line density gratings (e.g., 1800 lines/mm or 2400 lines/mm) provide higher spectral resolution but weaker signal intensity, while lower line density gratings (e.g., 600 lines/mm) yield stronger signal intensity but lower spectral resolution. For detectors, high-sensitivity CCD detectors, especially back-illuminated or deep-cooled CCDs, are preferred as they help reduce thermal noise.

### 3.2. Surface-Enhanced Raman Spectroscopy (SERS)

The instrumentation for SERS is similar to that of conventional Raman spectroscopy, with the key difference being the use of a nanostructured metallic substrate, such as silver or gold nanoparticles, in the sample preparation (Figure 4b,c). These metallic nanostructures enhance the local electromagnetic field through plasmonic effects, significantly amplifying the Raman signal. For SERS, it is important to select an appropriate excitation wavelength that matches the plasmon resonance wavelength of the substrate material (e.g., gold or silver) and its structure to achieve maximum signal enhancement. It is worth noting that higher laser power can produce stronger signals, but excessive power may cause thermal damage to the sample or substrate. Additionally, due to the small size of metallic nanostructures, objectives with a large numerical aperture are required to achieve precise focusing at the nanoscale. For asymmetric metallic nanostructured substrates, selecting the appropriate laser incidence angle and collection angle is critical for optimal signal acquisition.

### 3.3. Tip-Enhanced Raman Spectroscopy (TERS)

The instrumentation for TERS differs significantly from conventional Raman and SERS systems. TERS combines Raman spectroscopy with scanning probe systems, such as scanning tunneling microscopy (STM) or atomic force microscopy (AFM). The scanning probe system enables precise positioning of the material, while the strong localized electromagnetic field formed between the probe tip and the sample significantly enhances the Raman signal (Figure 4d). During TERS characterization, the laser is focused onto the probe tip through a microscope or a dedicated coupling optical path, and the scattered light is collected simultaneously. The size and shape of the probe tip are critical for TERS characterization [65]. Ultra-sharp tips can be obtained through chemical etching [66], or specific tip shapes can be fabricated using focused ion beam (FIB) or Ar^+^ ion sputtering techniques [67,68,69]. TERS systems are highly precise, and during TERS characterization, it is essential to ensure thermal insulation and vibration isolation of the optical platform and sample stage. This prevents optical path deviations and tip position shifts or state changes caused by mechanical vibrations or thermal effects.

### 3.4. Calibration

It is important to calibrate the instrument before performing Raman characterization (conventional Raman, SERS, or TERS). Calibration includes wavelength calibration, laser power calibration, spectral resolution calibration, and baseline calibration to ensure the accuracy of Raman shifts, spectral intensity, and resolution [62].

## 4. Conventional and Resonance-Enhanced Raman Spectroscopy Characterization

Since Raman spectroscopy fundamentally identifies the vibrational–rotational energy levels of molecules, its primary and most common application is in distinguishing and analyzing chemical bonds within materials. Raman spectroscopy proves particularly useful in the study of fullerene derivatives in the following ways: (1) The spherical structure of fullerene molecules gives rise to distinct Raman peaks, where shifts in peak positions and changes in intensity act as indicators of subtle structural modifications. Various functional groups introduce specific vibrational modes in Raman spectra, serving as “fingerprints” for chemical modifications, thus aiding in the precise identification and analysis of the chemical structures of fullerene derivatives. This provides direct evidence for understanding the impact of derivatives. By analyzing the vibrational modes and their variations, such as shifts in peak positions and intensity changes in Raman spectra, valuable insights into the electronic structure and charge distribution of molecules can be obtained. This is vital for comprehending the optoelectronic properties of fullerene derivatives, providing essential information on the intermolecular interactions of these derivatives. (2) Detecting charge transfer processes: When charge transfer occurs between fullerene and solutions or endohedral molecules, distinct Raman shifts arise due to changes in the vibration frequency of chemical bonds. By tracking and analyzing frequency shift, the conditions for charge transfer and the number of transferred electrons can be obtained. (3) Assessing environmental impact: Raman spectroscopy can evaluate how environmental factors affect fullerene derivatives. By recording the Raman spectra of molecules under varying solvent or temperature conditions, changes in molecular vibrational modes can be observed, allowing for the assessment of environmental factors’ influence on molecular structure and properties. Interactions between derivatives and solid substrates can also be characterized. Particularly, when molecules are in aqueous solution, which is general in biomedical research, the application of infrared spectroscopy is strongly restricted, whereas Raman spectrum is unlimited due to water’s weak Raman scattering intensity.

To achieve higher detection sensitivity, resonance-enhanced Raman spectroscopy can be utilized for the characterization of fullerenes. Resonance-enhanced Raman spectroscopy is a sophisticated analytical method that harnesses the resonance effect: when the excitation light frequency aligns with a molecule’s electronic transition energy levels, Raman signals from vibration modes coupled to these electronic transitions exhibit dramatic enhancement. Through the strategic selection of excitation wavelengths, this approach amplifies targeted vibrational modes by up to 10⁶-fold compared to conventional Raman spectroscopy, thus enabling the precise extraction of both structural and electronic characteristics. The technique’s mode-specific amplification is particularly valuable for analyzing fullerene derivatives, providing researchers with deeper insights into the detailed vibrational characteristics of these complex molecules. In addition, resonance-enhanced Raman spectroscopy allows for detection at lower concentrations of samples, which is especially valuable in biological and environmental contexts, where the presence of fullerene derivatives may be present in trace amounts. Although resonance-enhanced Raman spectroscopy involves greater equipment requirements and operational complexity compared to conventional methods, its superior selectivity and sensitivity make it an indispensable tool for studying complex molecular systems, such as fullerene derivatives. It is worth noting that due to the rich energy level system of fullerene derivatives, the incident light in the visible range can often resonate with a certain energy level.

### 4.1. Interaction Between Functional Groups and Fullerenes

**Figure 5:**
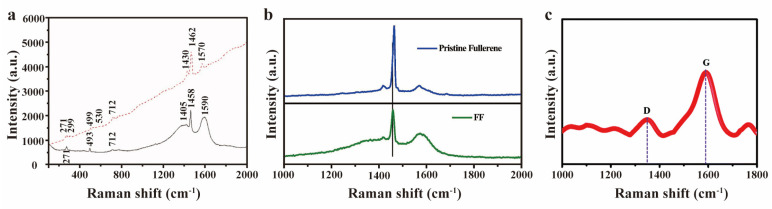
(**a**) Raman spectra of fullerene (solid line) and F-fullerene (dashed line). Reproduced with permission from [70]. Copyright 2011 John Wiley and Sons. (**b**) Raman spectra for fullerene before and after −SO_3_H group functionalization. Reproduced with permission from [50]. Copyright 2016 Elsevier. (**c**) Raman spectrum of the ethylenediamine functionalized C_60_. D and G represents D band and G band, respectively. Reproduced with permission from [71]. Copyright 2022 Royal Society of Chemistry.

Functionalized fullerenes exhibit broad applications across various fields, and investigating the binding interactions between functional groups and carbon cages serves as a fundamental scientific basis for tuning material properties. Raman spectroscopy’s high sensitivity to changes in molecular vibrational modes and structural configuration enables a detailed analysis of how functional groups alter fullerene cage geometry and electronic properties, while also uncovering the microscopic mechanisms that govern intramolecular interactions. Functional group addition causes structural deformation in fullerenes, manifesting as Raman peak shifts and intensity variations. These modifications introduce structural disorder and asymmetry, leading to peak broadening. Furthermore, the reduction in fullerene symmetry due to functionalization leads to the activation of previously forbidden Raman modes. The systematic analysis of these spectral changes—including peak position shifts, intensity modulations, and emergence of new modes—provides critical insights into structure–property relationships in functionalized fullerenes. D.-J. Chung et al. synthesized hydroxyl(-OH)-functionalized fullerene (F-fullerene) using γ-irradiation in a methanol/1,2-dichlorobenzene mixture solution [70]. The Raman features of this F-fullerene (Figure 5a) closely resembled those of the base fullerene, indicating that the fundamental structure was preserved. However, they noted shifts in peak positions from 1458 and 1590 cm^−1^ in the original fullerene to 1462 and 1570 cm^−1^, respectively, suggesting a slight distortion in the fullerene’s cage structure. Additionally, the disappearance of the fullerene D band and reduced intensity of the G band were attributed to the formation of a disordered sp^2^ carbon structure. G. Rambabu and colleagues used a similar analytical approach for fullerene functionalized with sulfonic acid (-SO_3_H) groups [50]. They observed a 6 cm^−1^ downshift in the pentagonal pinch mode A_g_(2) (1464 cm^−1^) upon functionalization (Figure 5b). Furthermore, the broadening of Raman peaks in F-fullerene was noted and attributed to the asymmetry introduced by surface functional groups. Recently, ethylenediamine functionalized fullerene was fabricated and characterized by S. S. Narwade et al. [71]. They identified a distinct G band at 1588.02 cm^−1^ (Figure 5c), which was attributed to the first-order scattering of the E_g_(2) mode for sp^2^ hybridization between the carbon atom in C_60_ and nitrogen atom in ethylenediamine, and a D band at 1344.71 cm^−1^ due to sp^3^-hybridized carbon and its structural defects (reflecting the introduced oxygen on fullerene forming O-C_60_ bond in this study). As functionalization progressed, they reported a D to G band intensity ratio (I_D_/I_G_) of 0.84, which reflects the degree of structural defects and sp^3^ carbon content in the functionalized fullerene. Martinez-Loran et al. functionalized fullerene thin films with 1,5-diaminonaphthalene [72], observing a decreased intensity of the primary Raman modes of C_60_ at 1427 (H_g_(7)), 1461 (A_g_(2)) and 1560 (H_g_(8)) cm^−1^, which illustrates the changes in the hybridization state (sp^2^ to sp^3^) of diamine-bound carbon atoms of fullerene. Similar phenomenon was also observed by Meza-Laguna et al. for 1,8-diaminooctane functionalization [73].

### 4.2. Interactions of Fullerene Derivatives with Solvents and Other Molecules

**Figure 6:**
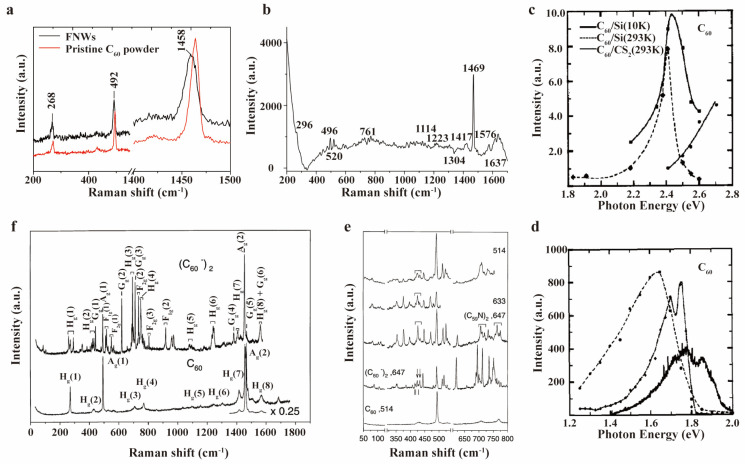
(**a**) Raman spectroscopy of FNWs and pristine C_60_ powder. Reproduced with permission from [74]. Copyright 2012 MDPI AG. (**b**) Raman spectrum of fullerene aqueous solution with 0.15 mg/mL concentration of C_60_ fullerene in water. Reproduced with permission from [75]. Copyright 2004 Elsevier. (**c**) Resonance−enhanced Raman spectrum for the 1469 cm^−1^ peak of C_60_ on silicon and dissolved in CS_2_, respectively. (**d**) Photoluminescence intensity of C_60_ excited with 5145 Å Ar^+^ laser line showing the minimum absorption level. C_60_ dissolved in CS_2_ at 293 K (thick solid line), deposited on Si at 293 K (dashed line) and at 10 K (thin solid line) are listed. (**c**,**d**) Reproduced with permission from [76]. Copyright 1991 Elsevier. (**e**) Resonance−enhanced Raman spectra of (C_59_N)_2_ and (C_60_)_2_ excited with different lasers in comparison with C_60_. (**f**) Resonance-enhanced Raman spectra of the (C_60_)_2_ dimer excited with 647.2 nm laser at 180 K in comparison with the spectra from C_60_ at room temperature. (**e**,**f**) Reproduced with permission from [77]. Copyright 2000 Springer Nature.

Interactions between fullerene derivatives and solvents or other molecules play a pivotal role in determining their properties and potential applications. Raman spectroscopy serves as a powerful analytical tool for monitoring structural modifications and property evolution during these interactions. Interactions between fullerene derivatives and solvents or other molecules can alter the electron cloud distribution, thereby influencing the chemical bond properties within the molecule. Since Raman scattering signals correspond to molecular vibrational modes, these electronic structure changes are manifested as shifts in Raman peak positions. In M. Sathish’s work, fullerene nanowhiskers showed an ~8 cm^−1^ downshift (Figure 6a) in the pentagonal pinch mode compared to powders, attributed to C_60_ polymerization [74]. In fullerene–water solutions, shifts of up to 15 cm^−1^ (Figure 6b) were observed for C_60_ Raman-active A_g_ and H_g_ bands, with additional vibrational bands at 1304 and 1637 cm^−1^ connected to the colloidal state of C_60_ [75]. K. Sinha et al. characterized a C_60_ film on a silicon surface and a solution of C_60_ in CS_2_ using resonance-enhanced Raman spectroscopy (Figure 6c) [76]. They analyzed excitation profiles (Raman intensity as a function of the laser photon energy) for A_g_(2) mode, and observed a peak around 2.4 eV in C_60_ thin film, suggesting optical transitions occurring well below the minimum absorption level anticipated for isolated icosahedral molecules. Moreover, this transition was not observed for C_60_ in solution. They proposed that solid-state effects might lower C_60_ symmetry, and thus permit HOMO to LUMO transitions (Figure 6d). W. Plank et al. used resonance-enhanced Raman spectroscopy to analyze single bonds between two C_60_ or C_59_N molecules and their influence on the molecules (Figure 6e,f) [77]. They observed strong resonances with red lasers, indicating the decreased electron transition energy positions in (C_59_N)_2_ and (C_60_)_2_ compared to individual C_60_ molecules, which was further confirmed by a lowered absorption edge in absorption spectroscopy. This decrease was attributed to intermolecular electron interactions in the dimer, which led to an electronic energy level redistribution.

**Figure 7:**
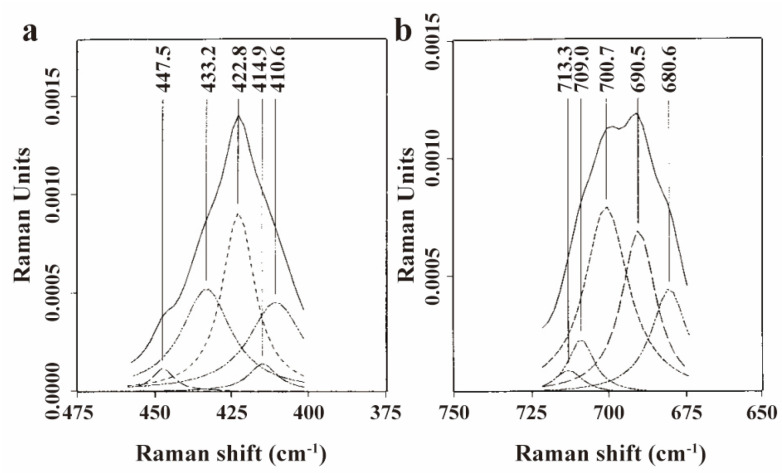
Raman spectra of (Ph_4_P)_2_·C_60_·Br H_g_(2) mode (**a**) and (Ph_4_P)_2_·C_60_·I H_g_(3) mode (**b**) excited with 1064 nm laser. Solid lines are experimental Raman data and dashed lines are fitted peaks with Lorentzian line shapes. (**a**,**b**) Reproduced with permission from [78]. Copyright 1999 Elsevier.

In 1999, M. Polomska and colleagues achieved resonance enhancement of low-frequency H_g_ modes of C_60_ tetraphenylphosphonium halides (Ph_4_P)_2_·C_60_·Y(Y = Cl, Br, I) single crystal (The complexes are classified under the triclinic space group, with each $C_{60}^{-}$ monoanion being encircled by eight phenyl rings of the (Ph_4_P)^+^ cations) using a 1064 nm laser, with modes broadened and downshifted due to electron–phonon interactions [78]. Resonance-enhanced spectroscopy’s high-resolution capability allowed the deconvolution of the H_g_ band into five components, which were attributed to decreased fullerene symmetry (Figure 7a,b). M. H. Herbst et al. employed resonance-enhanced Raman spectroscopy to trace the formation of the dimer and oligomer of platinum–fullerene compounds ([Pt_n_C_60_], where *n* = 1 and 2) [79]. The simultaneous observation of symmetric and asymmetric fullerene vibration modes in the spectrum indicates the formation of dimers and oligomers, respectively. The doublet splitting of the F_1u_(2) and F_1u_(4) mode also signaled the formation of an oligomer chain structure in these compounds.

### 4.3. Raman Investigation of Charge Transfer Properties in Fullerene Derivatives

**Figure 8:**
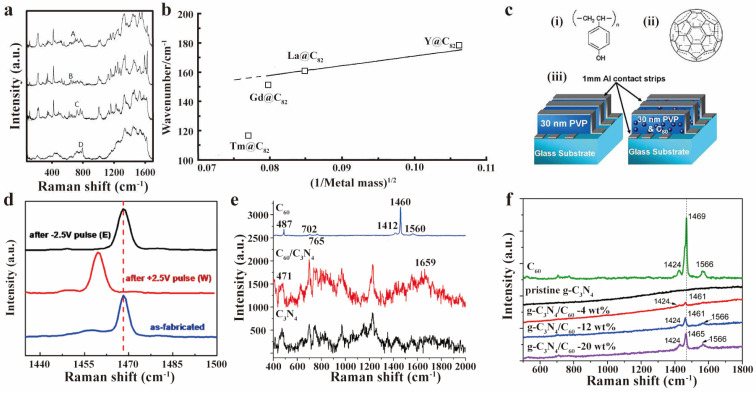
(**a**) Raman spectra of (A) Tm@C_82_, (B) Tm@C_82_, (C) TM@C_82_, and (D) La@C_82_ listed from top to bottom, respectively. (**b**) Plot of the wave number of the endohedral fullerenes Raman mode versus the square root of the reciprocal metal mass for Gd@C_82_, La@C_82_, Y@C_82_ and the three Tm@C_82_ isomers. (**a**,**b**) Reproduced with permission from [80]. Copyright 1999 Elsevier. (**c**) The structure of (**i**) PVP, (**ii**) C_60_, and schematic diagram of (**iii**) pure PVP MOM device with macroscopic cross point architecture and PVP + C_60_ device. (**d**) Raman spectra near the A_g_(1) C_60_ peak of a device after three memory operations. (**c**,**d**) Reproduced with permission from [85]. Copyright 2005 IOP Publishing. (**e**) Raman spectra of C_60_, g−C_3_N_4_ and C_60_/g−C_3_N_4_ photocatalysts. Reproduced with permission from [86]. Copyright 2014 Elsevier. (**f**) Raman spectroscopy of C_60_, pristine g−C_3_N_4_, g−C_3_N_4_/C_60_−4 wt%, g−C_3_N_4_/C_60_−12 wt% and g−C_3_N_4_/C_60_−20 wt% listed from top to bottom, respectively. Reproduced with permission from [87]. Copyright 2017 RSC Pub.

Charge transfer is a fundamental aspect of the involvement of fullerene derivatives in various chemical and physical processes. A precise examination of their charge transfer properties is crucial for understanding reaction mechanisms and regulating their functions. Raman spectroscopy, with its exceptional sensitivity to changes in molecular vibration frequencies, is a powerful tool for investigating the charge transfer properties of fullerene derivatives. The transferred electrons would alter vibrational frequencies of chemical bonds, leading to Raman peak shifts. By analyzing the trend of these frequency shifts, it is possible to determine the number of transferred electrons. M. Krause et al. embedded thulium and gadolinium into C_82_ molecular cages (Tm@C_82_ and Gd@C_82_, with Tm@C_82_ having three isomers A, B, and C) using a modified Krätschmer arc burning method, and characterized them with Raman spectroscopy (Figure 8a) [80]. They observed a band below 200 cm^−1^ (117 cm^−1^ for the isomers A and B, and 116 cm^−1^ for C) attributed to vibration between metal and cage, and the significant difference in Raman spectra between three isomers at 1550 to 1650 cm^−1^. In the plot of the wave number of the metal cage Raman mode below 200 cm^−1^ versus the square root of the reciprocal metal mass, Gd@C_82_, La@C_82_ and Y@C_82_ were found on one straight line, while three Tm@C_82_ isomers lowered significantly (Figure 8b). This was explained by differences in attractive forces between the metal ion, and the charged fullerene cage. They concluded that Gd@C_82_ correlates with trivalent ions as Gd^3+^@$C_{82}^{3-}$, similar to La^3+^ and Y^3+^. In contrast, Tm@C_82_ was associated with divalent ions as Tm^2+^@$C_{82}^{2-}$. In the same year, they also identified the molecule Eu@C_74_ to be Eu^2+^@$C_{74}^{2-}$ from analyzing Raman data [81]. Similar research was also conducted on endohedral fullerenes containing molecules such as La_2_@C_80_, Ti_2_@C_80_ [51], Gd_2_@C_79_N [82], Sc_3_N@C_80_ [83], Sc_4_N_2_@C_60_ [31] and U_2_@I_h_(7)-C_80_ [84]. S. Paul et al. and colleagues mixed C_60_ into insulating poly-vinyl-phenol (PVP) polymer and built a simple metal–organic–metal (MOM) sandwich structure device (Figure 8c) to test its memory effect [85]. Raman spectroscopy confirmed C_60_ involvement in charge storage. A downward shift in the A_g_(1) mode peak was observed after a +2.5 V write step, indicating charge injection into C_60_ molecules. The peak returned to 1469 cm^−1^ after a −2.5 V erase operation, signifying charge release (Figure 8d). Similar Raman techniques were used to analyze interactions between C_60_ and g-C_3_N_4_ [86,87]. Slight shifts in H_g_(7) and H_g_(8) modes, which correspond to higher-order vibrational modes of C_60_, indicated a charge transfer. The A_g_(2) mode disappeared when C_60_ was physically mixed with g-C_3_N_4_ (Figure 8e) [86], and showed a significant downshift when covalently bonded to g-C_3_N_4_ (Figure 8f) [87].

**Figure 9:**
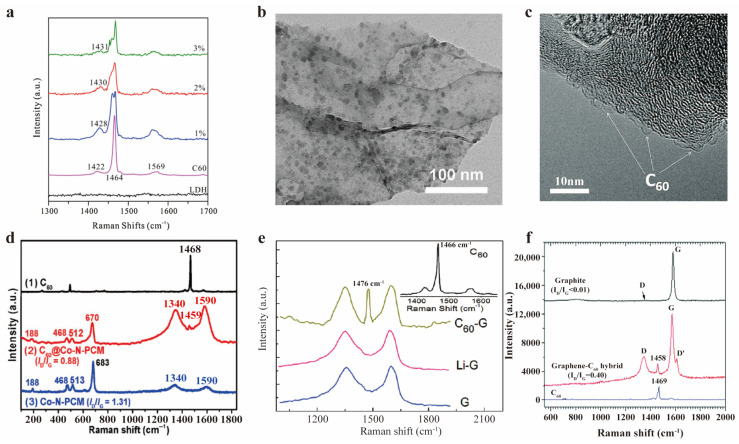
(**a**) Raman patterns of C_60_/LDH samples with various C_60_ contents. Reproduced with permission from [88]. Copyright 2017 Elsevier. (**b**) TEM images of the C_60_-grafted graphene. (**e**) Raman spectra of C_60_, the chemically reduced graphene (G), the lithiated graphene (Li-G), and the C_60_-grafted graphene (C_60_-G). (**b**,**e**) Reproduced with permission from [89]. Copyright 2011 American Chemical Society. (**c**) HR-TEM image of the graphene with C_60_ attached to edges. (**f**) Raman spectra of pristine graphite, graphene–C_60_ hybrid and C_60_. D and G represents D band and G band, respectively. (**c**,**f**) Reproduced with permission from [90]. Copyright 2015 Royal Society of Chemistry. (**d**) Raman spectra of C_60_, C_60_-embedded Co-N-PCM and Co-N-PCM. Reproduced with permission from [91]. Copyright 2020 Springer Nature.

**Figure 10:**
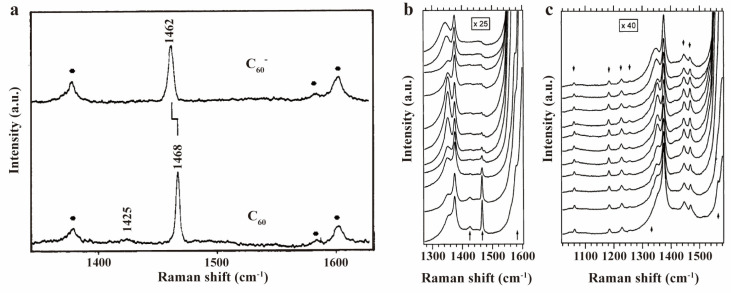
(**a**) Resonance−enhanced Raman spectra of $C_{60}$ and $C_{60}^{-}$ in (70%/30%) toluene/acetonitrile solvent. Reproduced with permission from [92]. Copyright 1993 American Chemical Society. (**b**,**c**) Raman spectra of C_60_@SWCNT and C_70_@SWCNT on Pt electrode, with the electrode potential varying by 0.3 V from −1.73 to 1.27 V vs. Fc/Fc^+^ for C_60_ ones and from −1.76 to 1.24 V vs. Fc/Fc^+^ for C_70_ ones from top to bottom. Arrows indicate the expected Raman lines of C_60_ and C_70_, respectively. (**b**,**c**) Reproduced with permission from [94]. Copyright 2005 Elsevier.

Interactions with layered double hydroxides (LDH) [88] resulted in electron transfer to C_60_, evidenced by upshifted Raman peaks (Figure 9a), which is beneficial for improving material’s catalytic ability. After covalent bonding with graphene, the A_g_(2) mode of C_60_ upshifted (Figure 9e) compared to pure molecules, indicating a strong interaction with graphene sheet [89]. However, when covalently attached to graphene nanoplatelet edges, the A_g_(2) mode exhibited an 11 cm^−1^ downshift (Figure 9f) [90]. Similarly, C_60_-embedded cobalt/nitrogen-codoped porous carbon materials (Co-N-PCM) also showed a downshifted A_g_(2) mode (Figure 9d), indicating the electron charge transfer to fullerene [91]. M. L. McGlashen and colleagues used resonance-enhanced Raman spectroscopy to characterize C_60_ monoanions in solution in 1993 [92]. They prepared C_60_ solutions in a 70%/30% toluene/acetonitrile mix and generated C_60_ monoanions by electrolysis at −1.0 V. The pentagonal pinch mode was strongly enhanced and showed a 6 cm^−1^ downshift compared to neutral molecules (Figure 10a). This is highly consistent with the shift in K-doped film data, thus highly supporting the hypothesis of complete electron transfer from K to C_60_. The structures of fullerene peapods, such as C_60_@single-walled carbon nanotubes (SWCNT), have become newly focused point in carbon nanomaterials research. L. Kavan et al. utilized the sensitivity of resonance-enhanced Raman spectroscopy to structures to characterize changes in fullerene caused by the outside SWCNT and anode/cathode charging [93], found that the A_g_(2) mode of C_60_ in fullerene peapods showed considerable intensity increase upon anodic doping (Figure 10b), which was not seen with cathodic charging. This differed from previously observed charging-induced peak shifts and was unique to fullerene peapods. They conducted follow-up experiments two years later, preparing another kind of fullerene peapods, C_70_@SWCNT, and characterizing it with resonance-enhanced Raman spectroscopy [94]. Unlike C_60_, C_70_@SWCNT exhibits symmetric quenching of the C_70_ Raman modes at both cathodic and anodic potentials (Figure 10c), indicating that the electronic structure of C_70_ responds differently to charging compared to C_60_. These experiments consistently highlight the charge transfer characterization capabilities of Raman spectroscopy in fullerene derivatives.

### 4.4. Resonance Enhancement Response of Fullerene Derivatives to Laser Wavelengths

**Figure 11:**
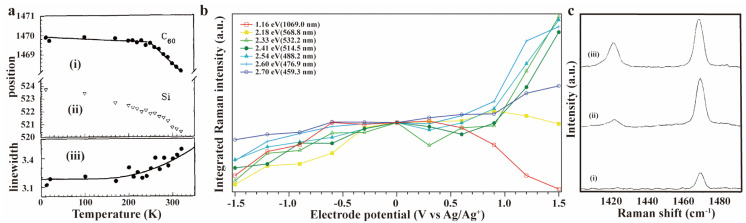
(**a**) Resonance−enhanced Raman line position of (i) C_60_ and (ii) silicon, and (iii) Raman line width of C_60_ as a function of temperature. Dots and triangles are experimental data. The full line drawn in (iii) is a calculation of two−phonon decay process. Reproduced with permission from [95]. Copyright 1991 Elsevier. (**b**) Normalized integrated Raman intensity of the A_g_(2) mode of C_60_ in SWCNT at different electrode potentials. Reproduced with permission from [96]. Copyright 2010 American Chemical Society. (**c**) Resonance−enhanced Raman spectra of C_60_ under the excitation wavelength of (i) 457.94, (ii) 413.10, and (iii) 406.67 nm, respectively. Reproduced with permission from [97]. Copyright 1994 American Chemical Society.

**Figure 12:**
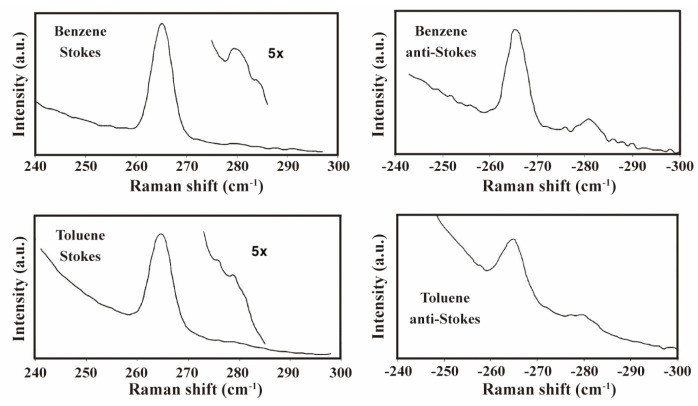
Stokes and anti−Stokes resonance-enhanced Raman spectrum of C_60_ under 413 nm excitation in benzene and toluene solvent, respectively. Reproduced with permission from [98]. Copyright 2004 American Chemical Society.

As previously mentioned, when the excitation wavelength matches the electronic transition energy levels, the Raman signals of specific vibrational modes associated with the electronic excited states are significantly enhanced. By investigating Raman spectral variations in fullerene materials under different excitation wavelengths, researchers can gain deeper insights into multiple characteristics including molecular structural properties, electronic behaviors, and intermolecular interactions. In 1991, M. Matus and colleagues conducted one of the first resonance-enhanced Raman characterizations of a mixture containing 85% C_60_ and 15% C_70_ on a silicon substrate [95]. They obtained Raman spectra using various excitation lasers with energies ranging from 3.04 to 1.90 eV (407.9 to 652.6 nm) at temperatures between 10 K and 320 K. A notable resonance enhancement in the C_60_ pinch mode at 1468 cm^−1^ was observed, with an enhancement factor of 100 between 2.0 eV (620.0 nm) and 2.6 eV (476.9 nm). Comparing the resonance-enhanced Raman peaks with theoretical predictions revealed two electronic transitions at 2.6 and 3.4 eV. They also noted a distinct inflection point (Figure 11a) in the temperature dependence of the Raman line position and width of the pinch mode. This was attributed to the phase transition. M. Kalbac et al. used laser with seven different excitation energies for resonance-enhanced Raman spectroscopy measurements to study A_g_(2) mode of C_60_ in SWCNT [96]. The A_g_(2) mode intensity weakened only under 1.16 eV (1069.0 nm) laser illumination, while increasing with other energies (2.70, 2.60, 2.54, 2.41, 2.33, 2.18 eV or 459.3, 476.9, 488.2, 514.5, 532.2, 568.8 nm) (Figure 11b). S. H. Gallagher et al. achieved resonance with the C_60_ A_1_(0–1) transition using a 406.7 nm laser and attained a higher H_g_ band intensity compared to the intensity excited with a 413.1 nm laser, which resonated with the A_0_(0–0) transition (Figure 11c) [97]. The intensity ratio of I_A1_/I_A0_, where I_A1_ and I_A0_ are the intensities of the H_g_ band at 1421 cm^−1^ excited from resonances with the A_1_ and A_0_ electronic transitions, matched the calculated value for D-term scattering within experimental error. Given that D-term scattering is a type of non-adiabatic scattering, this indicates significant non-adiabatic dynamic coupling between certain excited electronic states of the C_60_ molecule. In another study conducted by S. H. Gallagher et al., a resonance-enhanced Raman peak at 281 cm^−1^ also followed the 0–1 absorption envelope [98]. Surprisingly, the anti-Stokes side of this peak had greater intensity than the Stokes region under 413 nm laser irradiation in benzene or toluene solvent (Figure 12). This occurred despite thermal populations of the first vibrational excited state not exceeding 20%. Recently, F. Khorobrykh and coworkers observed similar phenomena, finding that varying the wavelength of exciting lasers could distinguish C_60_ clusters owning covalent bonds with different force constants [99]. In their works, higher force constant values corresponded to shorter wavelength excitation (405 nm in their work) being required for resonant Raman scattering observation.

The sensitivity of conventional and resonance-enhanced Raman spectroscopy to chemical bond vibrations and vibrational changes enables the detailed analysis of the microstructural changes, intermolecular interactions, and charge transfer mechanisms of fullerene derivatives. This capability provides critical insights into the behavior of fullerene derivatives in diverse environments. Additionally, due to the weak Raman scattering of water molecules, Raman spectroscopy is particularly well-suited for characterizing fullerene-related substances in solutions, making it valuable in biomedical research.

## 5. Surface-Enhanced Raman Spectroscopy Characterization

Surface-enhanced Raman spectroscopy (SERS) dramatically amplifies Raman signal intensity by exciting scattering from samples adsorbed onto nanostructured metal surfaces (e.g., gold, silver, or copper), achieving single-molecule detection limits and expanding Raman spectroscopy’s applications. Two main theories are widely accepted to explain this enhancement: localized surface plasmon resonance (LSPR) and charge transfer (CT) [63]. LSPR arises when incident light interacts with subwavelength metallic nanostructures, generating resonant electron oscillations that produce intense localized electric fields. These fields amplify Raman scattering from molecules within the near-field region. Concurrently, CT mechanisms, involving electron transitions between the metal and adsorbate, contribute through chemical enhancement. Together, these physical (LSPR) and chemical (CT) effects synergistically enhance SERS signals. SERS technology has the advantages of high sensitivity and non-destructiveness, making it an ideal tool for studying changes in the molecular structure of fullerene derivatives (such as the amorphization process) and intermolecular electronic coupling.

### 5.1. Advancements in SERS Characterization of C_60_: From Surface Interactions to Single-Molecule Detection

**Figure 13:**
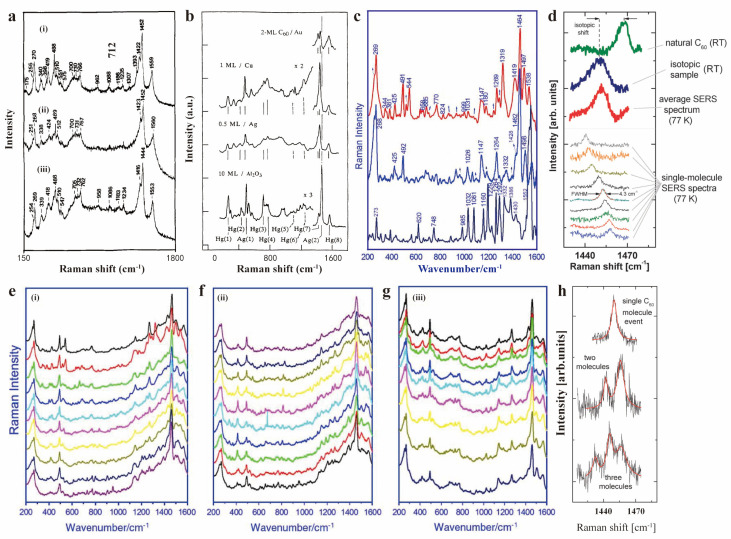
(**a**) SERS spectra of C_60_ molecules on an Au electrode in (i) pure water with no applied potential, (ii) 0.1 M aqueous KCl with +0.2 V vs. SCE, and (iii) 0.1 M KCl with −0.6 V vs. SCE. Reproduced with permission from [100]. Copyright 1991 American Chemical Society. (**b**) SERS spectra of C_60_ on Au, Cu, Ag and Al_2_O_3_ substrates. Reproduced with permission from [101]. Copyright 2024 American Physical Society. (**c**) SM−SERS of C_60_ from three different hot spots. (**e**–**g**) Time−dependent SM−SERS spectral fluctuations of C_60_ on Au−coated cover glass at three different hot spots (i), (ii) and (iii). (**c**,**e**–**g**) Reproduced with permission from [52]. Copyright 2011 John Wiley and Sons. (**d**) The top three lines show Raman spectra of natural and isotopic C_60_ molecules at room temperature, and an average SERS spectrum under 77 K. Bottom lines show different examples of SM−SERS signals. (**h**) SERS spectra of different number of C_60_ molecules. Fitting curves are shown as red lines. (**d**,**h**) Reproduced with permission from [53]. Copyright 2012 Royal Society of Chemistry.

Several research studies have been carried out on the high-sensitivity SERS characterization of fullerene materials. Early in 1991, R. L. Garrell and partners used SERS to characterize the structure and surface interactions of C_60_ on a gold surface [100]. Twenty-two distinct SERS bands were observed in the spectrum, far exceeding the number of modes in conventional Raman (Figure 13a). The newly observed bands were attributed to metal–molecule vibrations and reduced symmetry due to metal surface interactions, supported by the lower shifts in major C_60_ bands in SERS compared to conventional Raman. The next year, S. J. Chase et al. characterized the A_g_(2) band shift of C_60_ on different noble metal surfaces [101]. The largest shift of 28 cm^−1^ compared to pure C_60_ was observed for C_60_/Ag, followed by 23 cm^−1^ for C_60_/Cu, and the smallest of 15 cm^−1^ for C_60_/Au (Figure 13b). The large shift follows the decrease in noble metal work function, indicating a lower degree of charge transfer from metal to C_60_, which was further confirmed with the assistance of ultraviolet photoemission spectroscopy (UPS). L. Zhixun et al. observed single-molecule SERS (SM-SERS) behavior in samples with ultralow concentration solutions (10^−15^ M) of pyridine/C_60_ was dropped on Au-coated cover glass (Figure 13c) [52]. At this density, the statistical probability of a hot spot containing a single C_60_ molecule is less than one in a thousand. However, only around one-tenth of the intensity was observed for SM-SERS compared to the signal taken from the other sample with a 13-fold higher concentration (10^−2^ M). Several additional Raman bands were also observed and were attributed to the symmetry reduction and perturbation of electronic structure in the adsorbed single molecule. Upon serious observation of SM-SERS at different hot spots, some showed distinct band shifts, while the others had no change. Time-dependent spectral fluctuations of the SM-SERS taken at the same hot spot also reflect changes in intensity and band positions (Figure 13e–g). These observations collectively reflect that the SM-SERS achieved a much larger enhancement factor at some specific position. The exact SM-SERS was confirmed by C. G. Artur et al. in 2012 [53]. They differentiated SERS signals by using C_60_ molecules with different carbon isotopes (Figure 13d,h). By carefully screening the data and analyzing SERS spectra containing different numbers of molecules, the signal corresponding to the single molecule was successfully extracted, and the intensity and frequency distribution were consistent with theoretical expectations, indicating that single molecule events were, indeed, observed. These studies not only confirmed single-molecule detection capabilities, but also revealed symmetry alterations and electronic structure modifications of molecules localized within plasmonic hot spots.

### 5.2. Nanostructure Designs for Enhanced SERS of Fullerenes

To improve the detection sensitivity, researchers have designed and optimized nanostructures including gold/silver nanoparticles, core–shell nanorods and silver porous silicon, which have significantly boosted the SERS signal. L. Zhixun and F. Yan inserted the C_60_/C_70_ molecule into the gap between gold nanoparticles and the iron substrate, using the pyridine as an intermediate [59], and reached an enhancement factor of 10^6^. The next year, they achieved SERS enhancement by depositing gold/C_60_ (/C_70_) nanoclusters on the anodic alumina oxide nanosieves [102], as well as constructing a newly nonaqueous colloidal system [103]. Three years later, L. Zhixun, Z. Yongsheng and coworkers fabricated high-density ordered arrays of the core–shell nanopillars of Au@C_60_/C_70_ systems, which achieved an intense SERS signal with a fluorescence-free background was achieved [104]. This phenomenon persisted even at 514 nm excitation wavelength, where conventional Raman spectroscopy of C_60_/C_70_ exhibits weak signals with low signal-to-noise ratios (SNR). N. Zhiqiang et al. connected C_60_ molecules between silver nanoparticles and silver film by using the pyridine as an intermediate [105]. Several vibrational modes were greatly enhanced and showed significant band splitting and frequency shifting, suggesting symmetry reduction and relaxation of selection rules for C_60_ in asymmetric environments. I. I. S. Lim et al. found that when assembled with 1-(4-methyl)-piperazinyl fullerene (MPF), larger-scale Au nanoparticles (30 nm) produce much stronger SERS intensity than smaller ones (11 nm), indicating the influence of metal particle size on SERS enhancement [106]. Recently, N. Khinevich et al. achieved SERS enhancement of C_60_ using silvered porous silicon (Ag/PS) for the first time [107]. These methods not only improve the sensitivity of SERS spectrum, but also expand the application of SERS technology in the study of fullerenes and their derivatives.

### 5.3. SERS Applications in Studying Fullerene Interactions and Charge Transfer Behavior

**Figure 14:**
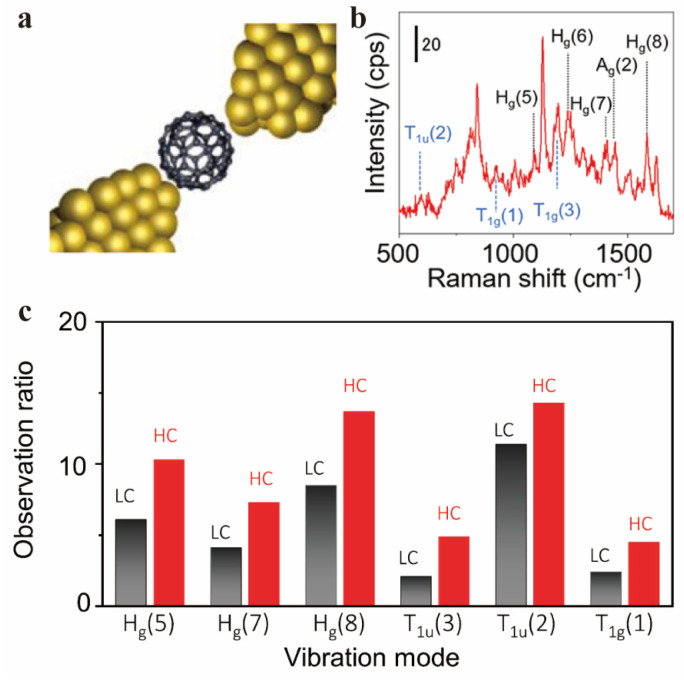
Single−molecule junction and SERS spectra in [111]. (**a**) Conceptual scheme of the C_60_ single−molecule junction. (**b**) SERS spectrum of the C_60_ SMJ at a conductance value of 5 × 10^−3^ G_0_ (G_0_ = 2e^2^/h). (**c**) Observation ratio of different vibrational modes under the LC and HC states, respectively. (**a**–**c**) Reproduced with permission from [111]. Copyright 2021 American Chemical Society.

The substantial signal amplification in surface-enhanced Raman spectroscopy (SERS) enables the precise characterization of interactions between fullerenes and other molecules or functional groups. Y. Zhao and Y. Fang studied interactions between C_60_ and pyridine molecules, confirming weak physical interactions due to unchanged C_60_ structures from SERS compared to the pure solid [108]. M. Baibarac et al. studied the particular chemical interaction of the polyaniline/fullerene (PANI/C_60_) composite with N-methyl-2-pyrrolidinone (NMP) using SERS [109]. During the interaction, de-doping of PANI was observed, which transformed leucoemeraldine salt (LS) repeating units into the leucoemeraldine base (LB). Additionally, a gradual increase in the intensity of the C_60_ A_g_(2) pentagonal pinch mode was also observed, which indicates the formation of PANI-LB and charge transfer complex of the type [(H)^+^(NMP)^+^(C_60_)^2−^]. N. Mojarad et al. used SERS to study the amorphization process of C_60_ molecules [110], as SERS is non-destructive and does not introduce external amorphization. All C_60_ resonances were suppressed under the electron irradiation with the appearance of G peak, which represents bond stretching of all pairs of sp^2^ hybridized atoms. The observation represents the cleavage of C_60_ bonds and the formation of a new structure. K. Yasuraoka and colleagues constructed a single-molecule junction by placing a C_60_ molecule between Au electrodes (Figure 14) [111]. The SERS spectrum showed an enhanced observation opportunity under the highly conductive state, where the molecular orbital and the Au electronic state are intensely coupled, suggesting that high electron coupling states benefit SERS observation. These studies uncovered the weak physical and chemical interactions, as well as the charge transfer behavior of fullerene by analyzing peak shifts, intensity changes, and the appearance of new vibrational modes in the Raman signal.

SERS technology is crucial in the study of fullerenes and their derivatives. It offers a highly sensitive method for single-molecule detection and elucidates the interaction mechanisms between fullerene molecules and other molecules or functional groups. Additionally, by designing various enhancing environments, the range of applications for SERS technology has been broadened.

## 6. Tip-Enhanced Raman Spectroscopy Characterization

Tip-enhanced Raman spectroscopy (TERS), a near-field spectroscopy that surpasses the optical diffraction limit, has shown unique advantages in characterizing single molecules in recent years. TERS focuses incident light onto a nanoscale (typically ranges from 10 to 100 nanometers) metal tip (typically gold or silver). It utilizes the local surface plasmon resonance effect, resulting in a strong electromagnetic field enhancement that significantly boosts the detection sensitivity of Raman signals [112]. The plasmonic enhancement effect in tip-enhanced Raman spectroscopy (TERS) empowers single-molecule vibrational mode detection, achieving a sub-nanometer spatial resolution [113] and enabling atomic-scale investigations of molecular conformations and localized chemical environments. Tip-enhanced Raman spectroscopy (TERS) offers transformative potential for fullerene research, combining sub-nanometer spatial resolution with signal-enhanced vibrational mode detection to enable the real-time, non-destructive tracking of structural and electronic dynamics in fullerene derivatives.

### 6.1. Advancements in TERS Characterization of Fullerene Molecules at the Nanoscale

**Figure 15:**
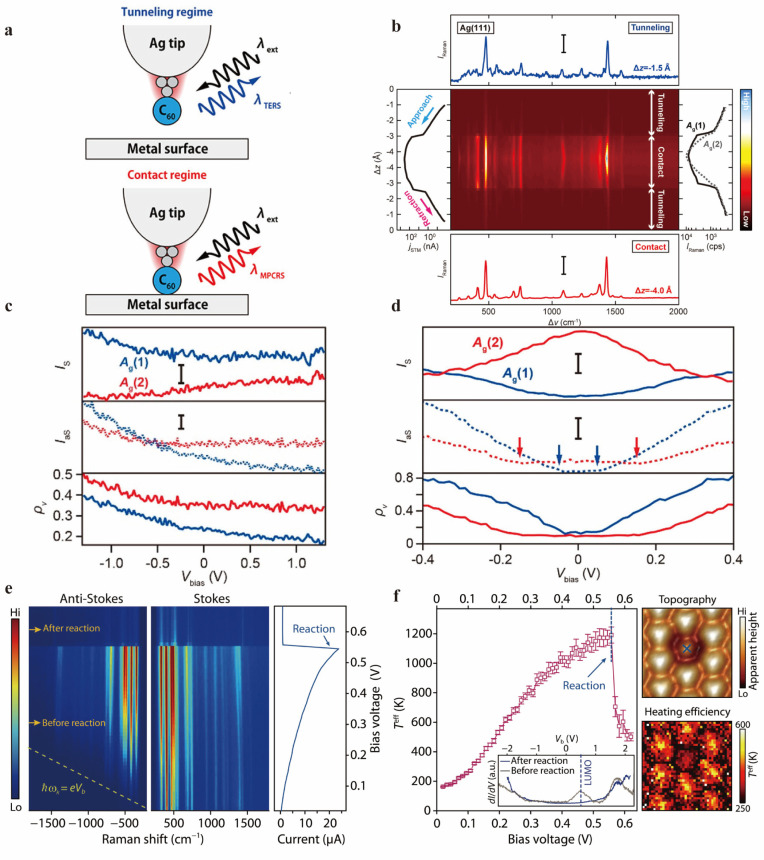
(**a**) Conceptual scheme of TERS under tunneling and MPC situations, respectively. (**b**) Δz−dependent single C_60_ TERS spectra measured on Ag(111) surface. The left panel shows the simultaneously obtained jSTM−Δz curve. The right panel shows the intensity of Ag modes as a function of the Δz. The top and bottom panels show the TERS spectra in the tunneling and MPC regime, respectively. (**a**,**b**) Reproduced with permission from [64]. Copyright 2022 American Chemical Society. (**c**,**d**) I_S_, I_aS_ and ρ_ν_ (ρ_ν_ ~ exp(−ħω_ν_/kT)) of the A_g_(1) and A_g_(2) modes as a function of V_bias_ under tunneling and MPC situation, respectively. Scale bar is 1000 cps for I_S_ and 500 cps for I_aS_. (**c**,**d**) Reproduced with permission from [117]. Copyright 2022 American Chemical Society. (**e**) Waterfall plot for the evolution of anti−Stokes and Stokes TERS spectra with increasing bias voltages under MPC situation. The right chart shows the simultaneously recorded tunneling currents. (**f**) Effective temperature as a function of bias voltage processed from (**e**). Inset graph shows dI/dV curves before and after decomposition. The right figures show STM topography (top) and corresponding effective temperature map (bottom, reconstructed from TERS spectrum) of the decomposed molecule(marked with a blue cross) surrounded by six intact C_60_. (**e**,**f**) Reproduced with permission from [118]. Copyright 2024 American Association for the Advancement of Science.

TERS technology offers significant advantages including high resolution and high sensitivity, prompting numerous research efforts aimed at optimizing the TERS characterization of fullerene materials. Early in 2000, R. M. Stöckle and coworkers had utilized TERS technology to characterize fullerene [114]. They drop-coated C_60_ molecules onto a glass substrate and scanned them with homemade tips fabricated by electrochemical etching with thin Au wire. A 488 nm laser was focused on the tip from below to excite the TERS. A C_60_ Raman signal was observed when the gold tip was close to the sample, with almost no signal detected without the tip. Considering the scale of the tip apex and laser beam diameter, the enhancement was estimated to be greater than 40,000. Both shifted and unshifted Raman peaks were observed, and were attributed to enhancement by electromagnetic and chemical effects, respectively. P. Verma et al. also achieved TERS enhancement of C_60_ using a silver-coated silicon tip and placed molecules on a silver film-covered glass cover slip [115]. All the Raman modes were enhanced in TERS signals compared to the far-field spectrum. In their subsequent investigation of the tip-force effect, the observed frequency shift caused by applying a force of 2.7 nN to 4.7 nN on C_60_ contradicted the predictions of DFT calculations. This phenomenon was attributed to photopolymerization under high tip pressure and under light irradiation at the same time. In recent years, advancements in TERS technology for single-molecule analysis have led to a surge in studies focused on the characterization of individual fullerene using TERS. B. Cirera and colleagues studied TERS intensities of a single C_60_ molecule in a scanning tunneling microscope (STM) system under tunneling regime and molecular point contact (MPC) situations, respectively (Figure 15a) [64]. They adsorbed C_60_ onto the Ag tip, and observed TERS signals as the distance between the molecule and Ag(111) surface varied. When the molecule contacted the metal surface, characteristic TERS intensity enhancement was observed, enabling the observation of modes undetectable in the tunneling regime (Figure 15b). A red shift in most C_60_ modes compared to solid-state values was also noted. These effects were similarly observed on other metal surfaces like Au(111), Cu(111), and Pt(111), confirming their universality. The additional enhancement in MPC situation was attributed to the chemical effects rather than electromagnetic enhancement, which was caused by charge transfer in the single molecule junction. B. Cirera et al. also achieved TERS enhancement with a factor of ~10^12^ for a C_60_ single molecule even on a Si(111)-(7 × 7) semiconductor surface, and observed additional Raman signal enhancement in MPC condition [116]. This indicates TERS’s applicability for characterizing organic molecules on semiconductor surfaces. It also shows potential for single-molecule Raman studies of vibrational heating and dissipation in metal–molecule–semiconductor nanoheterojunctions. These studies highlight the distinctive advantages of TERS in characterizing fullerenes and their derivatives at the single-molecule level. By integrating tip-enhancing technology with Raman spectroscopy, TERS can achieve significantly higher enhancement factors compared to traditional Raman spectroscopy (up to ~10^12^), which allows for the detection of the vibrational modes and chemical states of fullerene molecules at the nanoscale.

### 6.2. TERS for Investigating Local Thermal Effects of Single Fullerene Molecules

Leveraging its high spatial resolution, detection sensitivity, and in situ detection capabilities, TERS offers unique advantages for studying the local thermal effects of a single fullerene molecule with anti-Stokes TERS under non-equilibrium conditions. B. Cirera et al. conducted an MPC experiment to study vibrational heating effect by observing the anti-Stokes lines of C_60_ Raman modes [117]. They identified optical heating as the main mechanism in the tunneling regime (Figure 15c), while Joule heating dominated in MPC through inelastic electron–vibration scattering (IEVS) (Figure 15d). The influence of the electronic structure of the electrode to vibrational heating was also examined on different metal surfaces. The heating efficiency showed asymmetry in opposite bias polarity for Au(111) and Cu(111) surfaces and was lower when C_60_ was in point contact with the Pt(111) surface compared to other noble metals, although Pt exhibited the largest conductance. Q. Meng et al. also investigated the local thermal effect of a C_60_ single molecule via anti-Stokes TERS technology [118]. They studied the heating phenomenon with the defined effective temperature (T^eff^ ~ I_S_/I_aS_), as the tunneling current increased and observed the molecule decomposition at around 1150 K (Figure 15e,f). This study established TERS’s capability for non-invasive detection of localized thermal dynamics in single molecules under non-equilibrium conditions, while its spectral sensitivity to structural perturbations enabled the mechanistic elucidation of reaction pathways and intermediate states—a methodology extendable to diverse fullerene derivatives.

TERS technology not only offers a highly sensitive method for detecting molecular vibrational modes but also uncovers interactions between molecules and surfaces, as well as local thermal effects. These insights are crucial for understanding the electronic structure, chemical reactivity, and thermal stability of fullerene derivatives. However, TERS technology demands advanced experimental equipment and precise preparation of sharp tips. To further enhance detection sensitivity and spatial resolution, the development of more stable tips with higher enhancement factors is essential.

## 7. Conclusions and Perspectives

To date, Raman spectroscopy have provided distinct vibrational signatures that offer valuable insights into the molecular structure and electronic characteristics of fullerenes and their derivatives. Beyond structural elucidation, Raman spectroscopy also enables the investigation of fullerene–molecule interactions and environmental influences. By monitoring spectral variations in Raman peaks—including shifts, intensity changes, and linewidth broadening—under controlled experimental conditions, researchers can quantitatively evaluate how solvents, temperature, and other environmental parameters affect the structural and functional properties of fullerenes. Furthermore, advanced techniques such as resonance-enhanced Raman spectroscopy and SERS have significantly broadened the scope of fullerene research. Resonance-enhanced Raman spectroscopy enhances specific vibrational modes via resonance effects, while SERS facilitates single-molecule detection, thus providing deeper insights into fullerene structures and dynamics. TERS further pushes the frontier by enabling in situ analyses of fullerene derivatives under single-molecule conditions, delivering sub-nanometer spatial resolution and ultra-high detection sensitivity. Notably, TERS is also capable of detecting the optical and vibrational heating effects in individual molecule, as well as observing the thermal decomposition of molecule during the heating process.

Raman spectroscopy and its enhanced versions have already made significant progress in the study of fullerenes and their derivatives. In the following, we attempt to provide some possible future directions to further expand the application of Raman spectroscopy in this field.

### 7.1. Combining Raman Spectroscopy with Artificial Intelligence (AI)

The Raman spectra of fullerenes and their derivatives are relatively complex, and the orientation effects between the molecules and the SERS substrate further increase this complexity [119]. Machine learning is particularly well-suited for analyzing these intricate spectra, allowing for the extraction of key signals, identification of weak features, and elimination of unnecessary noise. Additionally, AI can be employed in the design of SERS substrates, potentially enhancing the detection efficiency of fullerene materials in SERS applications [120,121,122,123,124,125,126]. Developing machine learning algorithms and big data analytics to establish a comprehensive theoretical and experimental fullerene database will accelerate our understanding of fullerene systems and facilitate the discovery of new fullerene materials.

### 7.2. Further Improving the Resolution of TERS

TERS offers ultra-precise molecular characterization. Currently, angstrom-scale spatial resolution has been achieved with TERS on planar molecules such as tetraphenyl porphyrin (TPP) [127,128]. However, TERS studies on fullerene materials are primarily limited to C_60_, and the characterization of other fullerene derivatives remains relatively scarce. Achieving similar resolution for spherical fullerene structures presents additional challenges. Nevertheless, with ongoing advancements in TERS technology, attaining angstrom-scale spatial resolution for fullerene materials is likely within reach. Further improvements in the spatial resolution of TERS will enable the acquisition of more detailed structural and property information about fullerenes and their derivatives.

### 7.3. Combining Raman Spectroscopy with Time-Resolved Techniques

As femtosecond laser technology and ultrafast spectroscopic methods continue to mature, time-resolved Raman spectroscopy has been successfully applied to study ultrafast processes in various materials [129]. Examples include the ultrafast structural dynamics of photoreceptor proteins [130], the formation of polaron pairs and electron polarons in polymer photocatalysts [131], and the time dynamics of optical phonon anharmonic coupling and electron–phonon interactions in graphite [132,133]. Applying this technology to fullerene materials will enable the real-time characterization of structural and property changes in fullerene molecules and their derivatives during dynamic processes such as electrochemistry, catalysis, thermal decomposition, and light-induced transformations. This will provide essential data for advancing the preparation and application of fullerene-related materials.

## Figures and Tables

**Table 1:** Different Raman peaks of fullerene (cm^−1^).

Raman Spectra (C_60_)				Raman Spectra (C_70_)		
Symmetry				Symmetry		
	Meilunas et al. (1991) [55]	Vincenzo et al. (2001) [58]	Luo and Yan (2005) [59]		Bethune et al. (1991) [60]	Jishi et al. (1993) [61]
A_g_(1)	496	496	497	$A_{1}^{\prime }$		228
					459 *	455 *
A_g_(2)	1470	1468	1467		573 *	569 *
					739	737
H_g_(1)	273	264	273			1165
					1260	1257
H_g_(2)	434	430	433		1471	1469
				$E_{2}^{\prime }$	261	258
H_g_(3)	710	709	709		400	399
					411	409
H_g_(4)	774	773	772		501	508
					704	701
H_g_(5)	1100	1101	1100		1062	1062
					1186 *	1182 *
H_g_(6)	1250	1251	1249		1231 *	1227 *
					1298	1296
H_g_(7)	1426	1426	1422		1317	1313
					1336	1335
H_g_(8)	1576	1585	1574		1370	1367
					1448	1447
					1569	1565
				$E_{1}^{\prime \prime }$		714
					770 *	766 *
						1459
						1493
					1517 *	1515 *

* Raman peaks with uncertain vibration mode attribution.

## References

[1] Kroto H.W., Heath J.R., O’Brien S.C., Curl R.F., Smalley R.E. (1985). C_60_: Buckminsterfullerene. Nature.

[2] Olson J.R., Topp K.A., Pohl R.O. (1993). Specific Heat and Thermal Conductivity of Solid Fullerenes. Science.

[3] Cheng X., Yokozeki T., Yamamoto M., Wang H., Wu L., Koyanagi J., Sun Q. (2017). The decoupling electrical and thermal conductivity of fullerene/polyaniline hybrids reinforced polymer composites. Compos. Sci. Technol..

[4] Rustagi K.C., Nair S.V., Ramaniah L.M. (1997). Nonlinear optical response of fullerenes. Prog. Cryst. Growth Charact. Mater..

[5] Talapatra G.B., Manickam N., Samoc M., Orczyk M.E., Karna S.P., Prasad P.N. (1992). Nonlinear optical properties of the fullerene (C60) molecule: Theoretical and experimental studies. J. Phys. Chem..

[6] Billups W.E., Ciufolini M.A. (1993). Buckminsterfullerenes.

[7] Koruga D., Hameroff S.J.W.R.L., Sundareshan M. (1993). Fullerene C_60_: History, Physics, Nanobiology, Nanotechnology.

[8] Ehrenreich H., Spaepen F. (1994). Solid State Physics.

[9] Braun T., Schubert A., Maczelka H., Vasvári L. (1995). Fullerene Research 1985–1993.

[10] Dresselhaus M.S., Dresselhaus G., Eklund P.C. (1996). Science of Fullerenes and Carbon Nanotubes.

[11] Kroto H.W., Walton D.R.M. (1993). The Fullerenes: New Horizons for the Chemistry, Physics and Astrophysics of Carbon.

[12] Jiang Z., Zhao Y., Lu X., Xie J. (2021). Fullerenes for rechargeable battery applications: Recent developments and future perspectives. J. Energy Chem..

[13] Varma C.M., Zaanen J., Raghavachari K. (1991). Superconductivity in the Fullerenes. Science.

[14] Guo K., Li N., Bao L., Lu X. (2024). Fullerenes and derivatives as electrocatalysts: Promises and challenges. Green. Energy Environ..

[15] Pan Y., Liu X., Zhang W., Liu Z., Zeng G., Shao B., Liang Q., He Q., Yuan X., Huang D. (2020). Advances in photocatalysis based on fullerene C60 and its derivatives: Properties, mechanism, synthesis, and applications. Appl. Catal. B Environ..

[16] Ghavanloo E., Rafii-Tabar H., Kausar A., Giannopoulos G.I., Fazelzadeh S.A. (2023). Experimental and computational physics of fullerenes and their nanocomposites: Synthesis, thermo-mechanical characteristics and nanomedicine applications. Phys. Rep..

[17] Castro E., Garcia A.H., Zavala G., Echegoyen L. (2017). Fullerenes in biology and medicine. J. Mater. Chem. B.

[18] Pesado-Gómez C., Serrano-García J.S., Amaya-Flórez A., Pesado-Gómez G., Soto-Contreras A., Morales-Morales D., Colorado-Peralta R. (2024). Fullerenes: Historical background, novel biological activities versus possible health risks. Coord. Chem. Rev..

[19] Prato M. (1997). [60]Fullerene chemistry for materials science applications. J. Mater. Chem..

[20] Kerna N., Flores J. (2020). The Application of Fullerene Materials in Agriculture. EC Agric..

[21] Mukherjee A., Majumdar S., Servin A.D., Pagano L., Dhankher O.P., White J.C. (2016). Carbon Nanomaterials in Agriculture: A Critical Review. Front. Plant Sci..

[22] Haddon R.C., Brus L.E., Raghavachari K. (1986). Electronic structure and bonding in icosahedral C60. Chem. Phys. Lett..

[23] Xie S.-Y., Gao F., Lu X., Huang R.-B., Wang C.-R., Zhang X., Liu M.-L., Deng S.-L., Zheng L.-S. (2004). Capturing the Labile Fullerene [50] as C_50_Cl_10_. Science.

[24] Shinohara H., Sato H., Saito Y., Takayama M., Izuoka A., Sugawara T. (1991). Formation and extraction of very large all-carbon fullerenes. J. Phys. Chem..

[25] Prinzbach H., Weiler A., Landenberger P., Wahl F., Wörth J., Scott L.T., Gelmont M., Olevano D., Issendorff B. (2000). Gas-phase production and photoelectron spectroscopy of the smallest fullerene, C_20_. Nature.

[26] Parker D.H., Wurz P., Chatterjee K., Lykke K.R., Hunt J.E., Pellin M.J., Hemminger J.C., Gruen D.M., Stock L.M. (1991). High-yield synthesis, separation, and mass-spectrometric characterization of fullerenes C_60_ to C_266_. J. Am. Chem. Soc..

[27] Johnson R.D., de Vries M.S., Salem J., Bethune D.S., Yannoni C.S. (1992). Electron paramagnetic resonance studies of lanthanum-containing C_82_. Nature.

[28] Stevenson S., Burbank P., Harich K., Sun Z., Dorn H.C., van Loosdrecht P.H.M., deVries M.S., Salem J.R., Kiang C.H., Johnson R.D. (1998). La_2_@C_72_:  Metal-Mediated Stabilization of a Carbon Cage. J. Phys. Chem. A.

[29] Stevenson S., Rice G., Glass T., Harich K., Cromer F., Jordan M.R., Craft J., Hadju E., Bible R., Olmstead M.M. (1999). Small-bandgap endohedral metallofullerenes in high yield and purity. Nature.

[30] Korona T., Hesselmann A., Dodziuk H. (2009). Symmetry-Adapted Perturbation Theory Applied to Endohedral Fullerene Complexes: A Stability Study of H_2_@C_60_ and _2_H_2_@C_60_. J. Chem. Theory Comput..

[31] Wang T.-S., Chen N., Xiang J.-F., Li B., Wu J.-Y., Xu W., Jiang L., Tan K., Shu C.-Y., Lu X. (2009). Russian-Doll-Type Metal Carbide Endofullerene: Synthesis, Isolation, and Characterization of Sc_4_C_2_@C_80_. J. Am. Chem. Soc..

[32] Kurotobi K., Murata Y. (2011). A Single Molecule of Water Encapsulated in Fullerene C_60_. Science.

[33] Tóth É., Bolskar R.D., Borel A., González G., Helm L., Merbach A.E., Sitharaman B., Wilson L.J. (2005). Water-Soluble Gadofullerenes:  Toward High-Relaxivity, pH-Responsive MRI Contrast Agents. J. Am. Chem. Soc..

[34] Brettreich M., Hirsch A. (1998). A highly water-soluble dendro [60]fullerene. Tetrahedron Lett..

[35] Da Ros T., Prato M. (1999). Medicinal chemistry with fullerenes and fullerene derivatives. Chem. Commun..

[36] Zakharian T.Y., Seryshev A., Sitharaman B., Gilbert B.E., Knight V., Wilson L.J. (2005). A Fullerene−Paclitaxel Chemotherapeutic:  Synthesis, Characterization, and Study of Biological Activity in Tissue Culture. J. Am. Chem. Soc..

[37] Sitharaman B., Zakharian T.Y., Saraf A., Misra P., Ashcroft J., Pan S., Pham Q.P., Mikos A.G., Wilson L.J., Engler D.A. (2008). Water-Soluble Fullerene (C_60_) Derivatives as Nonviral Gene-Delivery Vectors. Mol. Pharm..

[38] Yin J.-J., Lao F., Fu P.P., Wamer W.G., Zhao Y., Wang P.C., Qiu Y., Sun B., Xing G., Dong J. (2009). The scavenging of reactive oxygen species and the potential for cell protection by functionalized fullerene materials. Biomaterials.

[39] del Carmen Gimenez-Lopez M., Räisänen M.T., Chamberlain T.W., Weber U., Lebedeva M., Rance G.A., Briggs G.A.D., Pettifor D., Burlakov V., Buck M. (2011). Functionalized Fullerenes in Self-Assembled Monolayers. Langmuir.

[40] Zaytseva O., Neumann G. (2016). Carbon nanomaterials: Production, impact on plant development, agricultural and environmental applications. Chem. Biol. Technol. Agric..

[41] Partha R., Conyers J.L. (2009). Biomedical applications of functionalized fullerene-based nanomaterials. Int. J. Nanomed..

[42] Bakry R., Vallant R.M., Najam-ul-Haq M., Rainer M., Szabo Z., Huck C.W., Bonn G.K. (2007). Medicinal applications of fullerenes. Int. J. Nanomed..

[43] Núñez-Regueiro M., Marques L., Hodeau J.L., Béthoux O., Perroux M. (1995). Polymerized Fullerite Structures. Phys. Rev. Lett..

[44] Rao A.M., Eklund P.C., Hodeau J.L., Marques L., Nunez-Regueiro M. (1997). Infrared and Raman studies of pressure-polymerized C_60_. Phys. Rev. B.

[45] Konarev D.V., Khasanov S.S., Saito G., Otsuka A., Yoshida Y., Lyubovskaya R.N. (2003). Formation of Single-Bonded (C_60_^−^)_2_ and (C_70_^−^)_2_ Dimers in Crystalline Ionic Complexes of Fullerenes. J. Am. Chem. Soc..

[46] Takashima A., Onoe J., Nishii T. (2010). In situ infrared spectroscopic and density-functional studies of the cross-linked structure of one-dimensional C_60_ polymer. J. Appl. Phys..

[47] Hou L., Cui X., Guan B., Wang S., Li R., Liu Y., Zhu D., Zheng J. (2022). Synthesis of a monolayer fullerene network. Nature.

[48] Hashikawa Y., Okamoto S., Murata Y. (2024). Synthesis of inter-[60]fullerene conjugates with inherent chirality. Nat. Commun..

[49] Hirsch A. (2010). The era of carbon allotropes. Nat. Mater..

[50] Rambabu G., Nagaraju N., Bhat S.D. (2016). Functionalized fullerene embedded in Nafion matrix: A modified composite membrane electrolyte for direct methanol fuel cells. Chem. Eng. J..

[51] Jaffiol R., Débarre A., Julien C., Nutarelli D., Tchénio P., Taninaka A., Cao B., Okazaki T., Shinohara H. (2003). Raman spectroscopy of La_2_@C_80_ and Ti_2_@C_80_ dimetallofullerenes. Phys. Rev. B.

[52] Luo Z., Loo B.H., Peng A., Ma Y., Fu H., Yao J. (2011). Single-molecule surface-enhanced Raman scattering of fullerene C_60_. J. Raman Spectrosc..

[53] Artur C.G., Miller R., Meyer M., Ru E.C.L., Etchegoin P.G. (2012). Single-molecule SERS detection of C_60_. Phys. Chem. Chem. Phys..

[54] Zhao Y., Luo X., Li H., Zhang J., Araujo P.T., Gan C.K., Wu J., Zhang H., Quek S.Y., Dresselhaus M.S. (2013). Interlayer Breathing and Shear Modes in Few-Trilayer MoS_2_ and WSe_2_. Nano Lett..

[55] Meilunas R., Chang R.P.H., Liu S., Jensen M., Kappes M.M. (1991). Infrared and Raman spectra of C_60_ and C_70_ solid films at room temperature. J. Appl. Phys..

[56] Sun G., Kertesz M. (2002). Vibrational Raman Spectra of C_70_ and C_706_^−^ Studied by Density Functional Theory. J. Phys. Chem. A.

[57] Dresselhaus M.S., Dresselhaus G., Eklund P.C. (1996). Raman Scattering in Fullerenes. J. Raman Spectrosc..

[58] Schettino V., Pagliai M., Ciabini L., Cardini G. (2001). The Vibrational Spectrum of Fullerene C_60_. J. Phys. Chem. A.

[59] Zhixun L., Yan F. (2005). SERS of gold/C_60_ (/C_70_) nano-clusters deposited on iron surface. Vib. Spectrosc..

[60] Bethune D.S., Meijer G., Tang W.C., Rosen H.J., Golden W.G., Seki H., Brown C.A., de Vries M.S. (1991). Vibrational Raman and infrared spectra of chromatographically separated C_60_ and C_70_ fullerene clusters. Chem. Phys. Lett..

[61] Jishi R.A., Dresselhaus M.S., Dresselhaus G., Wang K.-A., Zhou P., Rao A.M., Eklund P.C. (1993). Vibrational mode frequencies in C_70_. Chem. Phys. Lett..

[62] Long D.A. (2002). The Raman Effect: A Unified Treatment of the Theory of Raman Scattering by Molecules.

[63] Han X.X., Rodriguez R.S., Haynes C.L., Ozaki Y., Zhao B. (2022). Surface-enhanced Raman spectroscopy. Nat. Rev. Methods Primers.

[64] Cirera B., Litman Y., Lin C., Akkoush A., Hammud A., Wolf M., Rossi M., Kumagai T. (2022). Charge Transfer-Mediated Dramatic Enhancement of Raman Scattering upon Molecular Point Contact Formation. Nano Lett..

[65] Huang T.-X., Huang S.-C., Li M.-H., Zeng Z.-C., Wang X., Ren B. (2015). Tip-enhanced Raman spectroscopy: Tip-related issues. Anal. Bioanal. Chem..

[66] Ramanauskaite L., Xu H., Griskonis E., Batiuskaite D., Snitka V. (2018). Comparison and Evaluation of Silver Probe Preparation Techniques for Tip-Enhanced Raman Spectroscopy. Plasmonics.

[67] Liu S., Hammud A., Wolf M., Kumagai T. (2021). Anti-Stokes Light Scattering Mediated by Electron Transfer Across a Biased Plasmonic Nanojunction. ACS Photonics.

[68] Liu S., Cirera B., Sun Y., Hamada I., Müller M., Hammud A., Wolf M., Kumagai T. (2020). Dramatic Enhancement of Tip-Enhanced Raman Scattering Mediated by Atomic Point Contact Formation. Nano Lett..

[69] Fleischer M., Weber-Bargioni A., Altoe M.V.P., Schwartzberg A.M., Schuck P.J., Cabrini S., Kern D.P. (2011). Gold Nanocone Near-Field Scanning Optical Microscopy Probes. Acs Nano.

[70] Chung D.-J., Seong M.-K., Choi S.-H. (2011). Radiolytic synthesis of—OH group functionalized fullerene structures and their biosensor application. J. Appl. Polym. Sci..

[71] Narwade S.S., Mali S.M., Tanwade P.D., Chavan P.P., Munde A.V., Sathe B.R. (2022). Highly efficient metal-free ethylenediamine-functionalized fullerene (EDA@C_60_) electrocatalytic system for enhanced hydrogen generation from hydrazine hydrate. New J. Chem..

[72] Martinez-Loran E., Alvarez-Zauco E., Basiuk V.A., Basiuk E.V., Bizarro M. (2011). Fullerene thin films functionalized by 1,5-diaminonaphthalene: Preparation and properties. J. Nanosci. Nanotechnol..

[73] Meza-Laguna V., Basiuk E.V., Alvarez-Zauco E., Acosta-Najarro D., Basiuk V.A. (2007). Cross-linking of C_60_ films with 1,8-diaminooctane and further decoration with silver nanoparticles. J. Nanosci. Nanotechnol..

[74] Sathish M., Miyazawa K.I. (2012). Synthesis and Characterization of Fullerene Nanowhiskers by Liquid-Liquid Interfacial Precipitation: Influence of C_60_ Solubility. Molecules.

[75] Scharff P., Risch K., Carta-Abelmann L., Dmytruk I.M., Bilyi M.M., Golub O.A., Khavryuchenko A.V., Buzaneva E.V., Aksenov V.L., Avdeev M.V. (2004). Structure of C_60_ fullerene in water: Spectroscopic data. Carbon.

[76] Sinha K., Menéndez J., Hanson R.C., Adams G.B., Page J.B., Sankey O.F., Lamb L.D., Huffman D.R. (1991). Evidence for solid-state effects in the electronic structure of C_60_ films: A resonance-Raman study. Chem. Phys. Lett..

[77] Plank W., Pichler T., Kuzmany H., Dubay O., Tagmatarchis N., Prassides K. (2000). Resonance Raman excitation and electronic structure of the single bonded dimers (C ¯_60_)_2_ and (C _59_N)_2_. Eur. Phys. J. B Condens. Matter Complex. Syst..

[78] Polomska M., Sauvajol J.L., Graja A., Girard A. (1999). Resonant Raman scattering from single crystals (Ph_4_P)_2_·C_60_·Y. where Y = Cl, Br, I. Solid. State Commun..

[79] Herbst M.H., Pinhal N.M., Demétrio F.A.T., Dias G.H.M., Vugman N.V. (2000). Solid-state structural studies on amorphous platinum–fullerene [60] compounds [PtnC_60_] (n = 1, 2). J. Non-Cryst. Solids.

[80] Krause M., Kuran P., Kirbach U., Dunsch L. (1999). Raman and infrared spectra of Tm@C_82_ and Gd@C_82_. Carbon.

[81] Kuran P., Krause M., Bartl A., Dunsch L. (1998). Preparation, isolation and characterisation of Eu@C_74_: The first isolated europium endohedral fullerene. Chem. Phys. Lett..

[82] Fu W., Zhang J., Fuhrer T., Champion H., Furukawa K., Kato T., Mahaney J.E., Burke B.G., Williams K.A., Walker K. (2011). Gd_2_@C_79_N: Isolation, Characterization, and Monoadduct Formation of a Very Stable Heterofullerene with a Magnetic Spin State of S = 15/2. J. Am. Chem. Soc..

[83] Krause M., Kuzmany H., Georgi P., Dunsch L., Vietze K., Seifert G. (2001). Structure and stability of endohedral fullerene Sc_3_N@C_80_: A Raman, infrared, and theoretical analysis. J. Chem. Phys..

[84] Zhang X., Wang Y., Morales-Martínez R., Zhong J., de Graaf C., Rodríguez-Fortea A., Poblet J.M., Echegoyen L., Feng L., Chen N. (2018). U_2_@Ih(_7_)-C_80_: Crystallographic Characterization of a Long-Sought Dimetallic Actinide Endohedral Fullerene. J. Am. Chem. Soc..

[85] Paul S., Kanwal A., Chhowalla M. (2006). Memory effect in thin films of insulating polymer and C_60_ nanocomposites. Nanotechnology.

[86] Bai X., Wang L., Wang Y., Yao W., Zhu Y. (2014). Enhanced oxidation ability of g-C_3_N_4_ photocatalyst via C_60_ modification. Appl. Catal. B Environ..

[87] Chen X., Chen H., Guan J., Zhen J., Sun Z., Du P., Lu Y., Yang S. (2017). A facile mechanochemical route to a covalently bonded graphitic carbon nitride (g-C_3_N_4_) and fullerene hybrid toward enhanced visible light photocatalytic hydrogen production. Nanoscale.

[88] Zhu Y., Laipan M., Zhu R., Xu T., Liu J., Zhu J., Xi Y., Zhu G., He H. (2017). Enhanced photocatalytic activity of Zn/Ti-LDH via hybridizing with C_60_. Mol. Catal..

[89] Yu D., Park K., Durstock M., Dai L. (2011). Fullerene-Grafted Graphene for Efficient Bulk Heterojunction Polymer Photovoltaic Devices. J. Phys. Chem. Lett..

[90] Guan J., Chen X., Wei T., Liu F., Wang S., Yang Q., Lu Y., Yang S. (2015). Directly bonded hybrid of graphene nanoplatelets and fullerene: Facile solid-state mechanochemical synthesis and application as carbon-based electrocatalyst for oxygen reduction reaction. J. Mater. Chem. A.

[91] Wu J., Wang S., Lei Z., Guan R., Chen M., Du P., Lu Y., Cao R., Yang S. (2021). Pomegranate-like C_60_@cobalt/nitrogen-codoped porous carbon for high-performance oxygen reduction reaction and lithium-sulfur battery. Nano Res..

[92] McGlashen M.L., Blackwood M.E., Spiro T.G. (1993). Resonance Raman spectroelectrochemistry of the fullerene C_60_ radical anion. J. Am. Chem. Soc..

[93] Kavan L., Dunsch L., Kataura H. (2002). In situ Vis–NIR and Raman spectroelectrochemistry at fullerene peapods. Chem. Phys. Lett..

[94] Kavan L., Dunsch L., Kataura H. (2004). Electrochemical tuning of electronic structure of carbon nanotubes and fullerene peapods. Carbon..

[95] Matus M., Kuzmany H., Krätschmer W. (1991). Resonance Raman scattering and electronic transitions in C_60_. Solid. State Commun..

[96] Kalbac M., Zólyomi V., Rusznyák Á., Koltai J., Kürti J., Kavan L. (2010). An Anomalous Enhancement of the Ag(2) Mode in the Resonance Raman Spectra of C_60_ Embedded in Single-Walled Carbon Nanotubes during Anodic Charging. J. Phys. Chem. C.

[97] Gallagher S.H., Armstrong R.S., Lay P.A., Reed C.A. (1994). D-term scattering in the resonance Raman spectrum of C_60_. J. Am. Chem. Soc..

[98] Gallagher S.H., Thompson K.C., Armstrong R.S., Lay P.A. (2004). The Unusual Intensity Behavior of the 281-cm^−1^ Resonance Raman Band of C60:  A Complex Tale of Vibronic Coupling, Symmetry Reduction, Solvatochromism, and Jahn−Teller Activity. J. Phys. Chem. A.

[99] Khorobrykh F., Kulnitskiy B., Churkin V., Skryleva E., Parkhomenko Y., Zholudev S., Blank V., Popov M. (2022). The effect of C_60_ fullerene polymerization processes on the mechanical properties of clusters forming ultrahard structures of 3D C_60_ polymers. Diam. Relat. Mater..

[100] Garrell R.L., Herne T.M., Szafranski C.A., Diederich F., Ettl F., Whetten R.L. (1991). Surface-enhanced Raman spectroscopy of C_60_ on gold: Evidence for symmetry reduction and perturbation of electronic structure in the adsorbed molecule. J. Am. Chem. Soc..

[101] Chase S.J., Bacsa W.S., Mitch M.G., Pilione L.J., Lannin J.S. (1992). Surface-enhanced Raman scattering and photoemission of C_60_ on noble-metal surfaces. Phys. Rev. B.

[102] Zhixun L., Yan F., Pengxiang Z. (2006). Surface enhanced Raman scattering of gold/ C_60_ (/C_70_) nano-clusters deposited on AAO nano-sieve. Vib. Spectrosc..

[103] Luo Z., Fang Y. (2006). Investigation of the mechanism of influence of colloidal gold/silver substrates in nonaqueous liquids on the surface enhanced Raman spectroscopy (SERS) of fullerenes C_60_ (C_70_). J. Colloid. Interface Sci..

[104] Luo Z., Zhao Y.S., Yang W., Peng A., Ma Y., Fu H., Yao J. (2009). Core−Shell Nanopillars of Fullerene C60/C70 Loading with Colloidal Au Nanoparticles: A Raman Scattering Investigation. J. Phys. Chem. A.

[105] Niu Z., Fang Y. (2007). A new surface-enhanced Raman scattering system for C_60_ fullerene: Silver nano-particles/ C_60_ /silver film. Vib. Spectrosc..

[106] Lim I.I.S., Pan Y., Mott D., Ouyang J., Njoki P.N., Luo J., Zhou S., Zhong C.-J. (2007). Assembly of Gold Nanoparticles Mediated by Multifunctional Fullerenes. Langmuir.

[107] Khinevich N., Girel K., Bandarenka H., Salo V., Mosunov A. (2017). Surface enhanced Raman spectroscopy of fullerene C_60_ drop-deposited on the silvered porous silicon. J. Phys. Conf. Ser..

[108] Zhao Y., Fang Y. (2004). Fluorescence of C_60_ and Its Interaction with Pyridine. J. Phys. Chem. B.

[109] Baibarac M., Baltog I., Daescu M., Lefrant S., Chirita P. (2016). Optical evidence for chemical interaction of the polyaniline/fullerene composites with N-methyl-2-pyrrolidinone. J. Mol. Struct..

[110] Mojarad N., Tisserant J.-N., Beyer H., Dong H., Reissner P.A., Fedoryshyn Y., Stemmer A. (2017). Monitoring the transformation of aliphatic and fullerene molecules by high-energy electrons using surface-enhanced Raman spectroscopy. Nanotechnology.

[111] Yasuraoka K., Kaneko S., Kobayashi S., Tsukagoshi K., Nishino T. (2021). Surface-Enhanced Raman Scattering Stimulated by Strong Metal–Molecule Interactions in a C_60_ Single-Molecule Junction. ACS Appl. Mater. Interfaces.

[112] Pettinger B., Schambach P., Villagómez C.J., Scott N. (2012). Tip-Enhanced Raman Spectroscopy: Near-Fields Acting on a Few Molecules. Annu. Rev. Phys. Chem..

[113] Pozzi E.A., Goubert G., Chiang N., Jiang N., Chapman C.T., McAnally M.O., Henry A.-I., Seideman T., Schatz G.C., Hersam M.C. (2017). Ultrahigh-Vacuum Tip-Enhanced Raman Spectroscopy. Chem. Rev..

[114] Stöckle R.M., Suh Y.D., Deckert V., Zenobi R. (2000). Nanoscale chemical analysis by tip-enhanced Raman spectroscopy. Chem. Phys. Lett..

[115] Verma P., Yamada K., Watanabe H., Inouye Y., Kawata S. (2006). Near-field Raman scattering investigation of tip effects on C_60_ molecules. Phys. Rev. B.

[116] Cirera B., Liu S., Park Y., Hamada I., Wolf M., Shiotari A., Kumagai T. (2024). Single-molecule tip-enhanced Raman spectroscopy of C_60_ on the Si(111)-(7 × 7) surface. Phys. Chem. Chem. Phys..

[117] Cirera B., Wolf M., Kumagai T. (2022). Joule Heating in Single-Molecule Point Contacts Studied by Tip-Enhanced Raman Spectroscopy. Acs Nano.

[118] Meng Q., Zhang J., Zhang Y., Chu W., Mao W., Zhang Y., Yang J., Luo Y., Dong Z., Hou J.G. (2024). Local heating and Raman thermometry in a single molecule. Sci. Adv..

[119] Lussier F., Thibault V., Charron B., Wallace G.Q., Masson J.-F. (2020). Deep learning and artificial intelligence methods for Raman and surface-enhanced Raman scattering. TrAC Trends Anal. Chem..

[120] Bi X., Lin L., Chen Z., Ye J. (2024). Artificial Intelligence for Surface-Enhanced Raman Spectroscopy. Small Methods.

[121] Yi J., You E.-M., Liu G.-K., Tian Z.-Q. (2024). AI–nano-driven surface-enhanced Raman spectroscopy for marketable technologies. Nat. Nanotechnol..

[122] Cialla-May D., Bonifacio A., Markin A., Markina N., Fornasaro S., Dwivedi A., Dib T., Farnesi E., Liu C., Ghosh A. (2024). Recent advances of surface enhanced Raman spectroscopy (SERS) in optical biosensing. TrAC Trends Anal. Chem..

[123] Robles-Hernández J.-S.-L., Medina D.I., Aguirre-Hurtado K., Bosquez M., Salcedo R., Miralrio A. (2024). AI-assisted models to predict chemotherapy drugs modified with C_60_ fullerene derivatives. Beilstein J. Nanotechnol..

[124] Hu W., Ye S., Zhang Y., Li T., Zhang G., Luo Y., Mukamel S., Jiang J. (2019). Machine Learning Protocol for Surface-Enhanced Raman Spectroscopy. J. Phys. Chem. Lett..

[125] Liu Q., Yu W., Liu A., Chen Z., Xia X., Huang Y., Shi H. (2024). AI Algorithm-Assisted SERS Detection of Levothyroxine Sodium in Urine by an Optoplasmonic Film. J. Phys. Chem. C.

[126] Srivastava S., Wang W., Zhou W., Jin M., Vikesland P.J. (2024). Machine Learning-Assisted Surface-Enhanced Raman Spectroscopy Detection for Environmental Applications: A Review. Environ. Sci. Technol..

[127] Lee J., Crampton K.T., Tallarida N., Apkarian V.A. (2019). Visualizing vibrational normal modes of a single molecule with atomically confined light. Nature.

[128] Zhang Y., Yang B., Ghafoor A., Zhang Y., Zhang Y.-F., Wang R.-P., Yang J.-L., Luo Y., Dong Z.-C., Hou J.G. (2019). Visually constructing the chemical structure of a single molecule by scanning Raman picoscopy. Natl. Sci. Rev..

[129] Kuramochi H., Tahara T. (2021). Tracking Ultrafast Structural Dynamics by Time-Domain Raman Spectroscopy. J. Am. Chem. Soc..

[130] Kuramochi H., Takeuchi S., Yonezawa K., Kamikubo H., Kataoka M., Tahara T. (2017). Probing the early stages of photoreception in photoactive yellow protein with ultrafast time-domain Raman spectroscopy. Nat. Chem..

[131] Piercy V.L., Saeed K.H., Prentice A.W., Neri G., Li C., Gardner A.M., Bai Y., Sprick R.S., Sazanovich I.V., Cooper A.I. (2021). Time-Resolved Raman Spectroscopy of Polaron Formation in a Polymer Photocatalyst. J. Phys. Chem. Lett..

[132] Yang J.-A., Parham S., Dessau D., Reznik D. (2017). Novel Electron-Phonon Relaxation Pathway in Graphite Revealed by Time-Resolved Raman Scattering and Angle-Resolved Photoemission Spectroscopy. Sci. Rep..

[133] Yan H., Song D., Mak K.F., Chatzakis I., Maultzsch J., Heinz T.F. (2009). Time-resolved Raman spectroscopy of optical phonons in graphite: Phonon anharmonic coupling and anomalous stiffening. Phys. Rev. B.

